# Visualizing Volumetric and Segmentation Data using Mol* Volumes & Segmentations 2.0

**DOI:** 10.1002/cpz1.70070

**Published:** 2024-12-09

**Authors:** Aliaksei Chareshneu, Alessio Cantara, Dominick Tichý, David Sehnal

**Affiliations:** ^1^ National Centre for Biomolecular Research, Faculty of Science Masaryk University Brno Czech Republic; ^2^ Protein Data Bank in Europe, European Molecular Biology Laboratory European Bioinformatics Institute Hinxton Cambridge United Kingdom of Great Britain and Northern Ireland; ^3^ The authors wish it to be known that, in their opinion, the first two authors should be regarded as joint first authors

**Keywords:** 3D visualization tools, annotation data, large‐scale datasets, segmentation data, volumetric data

## Abstract

Ever‐increasing availability of experimental volumetric data (e.g., in .ccp4, .mrc, .map, .rec, .zarr, .ome.tif formats) and advances in segmentation software (e.g., Amira, Segger, IMOD) and formats (e.g., .am, .seg, .mod, etc.) have led to a demand for efficient web‐based visualization tools. Despite this, current solutions remain scarce, hindering data interpretation and dissemination. Previously, we introduced Mol* Volumes & Segmentations (Mol* VS), a web application for the visualization of volumetric, segmentation, and annotation data (e.g., semantically relevant information on biological entities corresponding to individual segmentations such as Gene Ontology terms or PDB IDs). However, this lacked important features such as the ability to edit annotations (e.g., assigning user‐defined descriptions of a segment) and seamlessly share visualizations. Additionally, setting up Mol* VS required a substantial programming background. This article presents an updated version, Mol* VS 2.0, that addresses these limitations. As part of Mol* VS 2.0, we introduce the Annotation Editor, a user‐friendly graphical interface for editing annotations, and the Volumes & Segmentations Toolkit (VSToolkit) for generating shareable files with visualization data. The outlined protocols illustrate the utilization of Mol* VS 2.0 for visualization of volumetric and segmentation data across various scales, showcasing the progress in the field of molecular complex visualization. © 2024 The Author(s). Current Protocols published by Wiley Periodicals LLC.

**Basic Protocol 1**: VSToolkit—setting up and visualizing a user‐constructed Mol* VS 2.0 database entry

**Basic Protocol 2**: VSToolkit—visualizing multiple time frames and volume channels

**Support Protocol 1**: Example: Adding database entry idr‐13457537

**Alternate Protocol 1**: Local‐server‐and‐viewer—visualizing multiple time frames and volume channels

**Support Protocol 2**: Addition of database entry custom‐tubhiswt

**Basic Protocol 3**: VSToolkit—visualizing a specific channel and time frame

**Basic Protocol 4**: VSToolkit—visualizing geometric segmentation

**Basic Protocol 5**: VSToolkit—visualizing lattice segmentations

**Alternate Protocol 2**: “Local‐server‐and‐viewer”—visualizing lattice segmentations

**Basic Protocol 6**: “Local‐server‐and‐viewer”—visualizing multiple volume channels

**Support Protocol 3**: Deploying a server API

**Support Protocol 4**: Hosting Mol* viewer with VS extension 2.0

**Support Protocol 5**: Example: Addition of database entry empiar‐11756

**Support Protocol 6**: Example: Addition of database entry emd‐1273

**Support Protocol 7**: Editing annotations

**Support Protocol 8**: Addition of database entry idr‐5025553

## INTRODUCTION

In the domain of biological research, volume segmentations—the results of the process of partitioning two‐dimensional (2D) or three‐dimensional (3D) images into regions (i.e., segments) corresponding to specific objects—serve as a critical bridge for interpreting imaging data within its biological context. The expanding availability of volume and segmentation datasets, deposited in public repositories because of advancements in segmentation tools (Kievits et al., 2022; Thomas & John, 2017), data format standardization (Patwardhan et al., 2017), and data‐sharing pipelines (Wilson et al., 2021), necessitates the development of appropriate visualization tools. Although desktop applications for segmentation data visualization are increasingly prevalent (Fedorov et al., 2012; Haberl et al., 2018; Humphrey et al., 1996; Lancaster et al., 2010; Peng et al., 2010; Pettersen et al., 2021), public repositories often lack such capabilities. This highlights the pressing need for interactive web‐based visualization tools specifically tailored for 3D volume segmentations.

However, the utility of segmentations is significantly diminished without annotations. Annotations, encompassing all relevant information about volume or segmentation data, can be categorized as either related to visual representation (e.g., specifying colors for volume channels or segments) or semantically relevant (e.g., providing information on biological function, cell type, or corresponding biological entities). For instance, annotations can contain information about the tissue affiliation or the cell type of a segmented cell. In the case of an organelle, annotations can emphasize its functions and involvement in biological processes. Additionally, annotations can incorporate references to external resources such as PRO (Natale et al., 2017), UniProt (The UniProt Consortium, 2023), or Gene Ontology (GO; Ashburner et al., 2000). Despite their value in enriching biological context, currently available software tools offer limited support for visualization of annotations alongside segmentation data.

To address these limitations, we previously developed Mol* Volumes & Segmentations (Mol* VS)—a web application designed for the visualization of large‐scale volumetric, segmentation, and annotation data (Chareshneu et al., 2023). Mol* VS offered functionalities such as displaying biological context annotations for each segment, supplementing data with macromolecular coordinates, enabling concurrent visualization of different segmentations for facilitating comparison, and a data streaming option.

Building upon the foundation established by Mol* VS, we present the next iteration of the application, Mol* VS 2.0, incorporating additional features. These enhancements include the ability to add, remove, or modify volumes, segmentations, and annotations alongside user control over downsampling procedures, providing the ability to limit the maximum and minimum downsampling level to reduce the size of the dataset. Additionally, support for widely used segmentation data formats, such as electron density masks and OME NGFF (Moore et al., 2021), has been implemented. To streamline the visualization of volume, segmentation, and annotation data, we have developed the Volumes & Segmentations Toolkit (VSToolkit), offering a novel and user‐friendly approach for visualizing the selected subset of database entry data. It provides an easy way for the user to both visualize a selected subset of data by specifying its content (specific volume channel, time frame, or segmentation) and share the resulting visualization (either via a link to publicly hosted file or via dragging‐and‐dropping the locally available file to the instance Mol* Viewer with Mol* VS 2.0 extension).

The protocols outlined herein demonstrate the practical application of Mol* Volumes & Segmentations 2.0, in conjunction with the VSToolkit, for generating information‐rich 3D visualizations of biomacromolecules across various scales. These protocols encompass a diverse range of use cases, including the visualization of protein complexes (e.g., the *Drosophila melanogaster* CMG helicase complex: database entry emd‐1832; see Basic Protocol 1 for details), cellular organelles (e.g., centrioles and Golgi apparatus: database entry emd‐1273, see Basic Protocols 5 and 6; or ribosomes, as particles: database entry empiar‐11756, see Basic Protocol 4), whole cells (e.g., fibroblasts: database entry idr‐13457537; see Basic Protocols 2 and 3), and tissues (e.g., tonsil sections: database entry idr‐5025553; see Basic Protocol 6). The application supports not only 3D data but also 2D data (see Basic Protocol 6). In addition, the protocols showcase the wide range of supported input formats, including but not limited to electron density maps (https://www.ebi.ac.uk/emdb/documentation#data_model; Basic Protocol 1), tomograms (Basic Protocols 4‐6), EMDB SFF (https://www.ebi.ac.uk/emdb/documentation#seg_model; Basic Protocol 1), OME NGFF (Basic Protocols 2 and 3), OME TIFF (Besson et al., 2019; Alternate Protocol 1), and electron density masks (Basic Protocol 5 and Alternate Protocol 2). Finally, we discuss here the two visualization approaches available in Mol* VS 2.0: one utilizes the VSToolkit in combination with a publicly available instance of the Mol* 3D viewer with Mol* VS 2.0 extension (https://molstar.org/molstar‐volseg/), whereas the other involves hosting local instances of the server API and Mol* 3D viewer (“local‐server‐and‐viewer” approach).

These protocols collectively highlight the significant advancements made in the visualization of complex molecular structures, coupled with the display of biologically relevant annotations. This enhanced visualization capability is expected to enhance our understanding of biological processes at various scales.

## Basic Protocol 1 VSToolkit—SETTING UP AND VISUALIZING A USER‐CONSTRUCTED Mol* VS 2.0 DATABASE ENTRY

This protocol provides a basic framework for the visualization of volumetric, segmentation, and annotation data with Mol* VS 2.0 (a database entry) and outlines the functionality of the VSToolkit, allowing users to generate a Cell* Volumes & Segmentations Archive (CVSX) file. CVSX format enables visualization within a publicly available instance of the Mol* 3D viewer with Mol* VS 2.0 extension (http://molstar.org/molstar‐volseg/). Notably, each step within this protocol is modular, allowing them to be combined to create more intricate visualizations. Furthermore, the actions described can be repeatedly applied to diverse source data, effectively facilitating user‐driven sharing and reuse of visualization snippets across protocols.

The primary objective of this protocol is the use of the VSToolkit to generate a CVSX file encapsulating both volume and segmentation data, along with biological annotations. This file can then be seamlessly opened and visualized within the Mol* 3D viewer. We have chosen the emd‐1832 dataset (https://www.ebi.ac.uk/emdb/EMD‐1832), with data for the *Drosophila melanogaster* CMG helicase complex, for this example because of its modest size, which will help the user to run this introductory example smoothly. From a biological point of view, it provides an insight into the process of initiating eukaryotic DNA replication with the focus on the structures of Mcm2‐Mcm7 and the CMG complex obtained using single‐particle electron microscopy.

### Necessary Resources

#### Hardware

Internet connection; device capable of supporting a Web browser, WebGL, and Python

#### Software

Python 3.10 (https://www.python.org/downloads/release/python‐3100/), mamba (https://mamba.readthedocs.io/en/latest/) or conda (https://conda.io/projects/conda/en/latest/user‐guide/install/index.html) and up to date Web browser (e.g., Google Chrome, Firefox, Apple Safari) with WebGL support

1 Install the server; set up and activate the environment:
  a. Clone the Github repository and change current directory to the root repository directory (molstar‐volseg by default):
git clone https://github.com/molstar/molstar‐volseg.git
cd molstar‐volseg
  b. Set up the environment:
  Option A. Using Conda package manager:
conda env create ‐f environment.yaml
  Option B. Using Mamba:
mamba env create ‐f environment.yaml
2 Activate the created environment:
conda activate cellstar‐volume‐server
3 Add EMD‐1832 (obtained from EMDB) raw data as an entry to the user‐constructed Mol* VS 2.0 database:
python preprocessor/cellstar_preprocessor/preprocess.py preprocess ‐‐mode add ‐‐input‐path test‐data/preprocessor/sample_volumes/emdb/EMD‐1832.map ‐‐input‐kind map ‐‐input‐path test‐data/preprocessor/sample_segmentations/emdb_sff/emd_1832.hff ‐‐input‐kind sff –entry‐id emd‐1832 ‐‐source‐db emdb ‐‐source‐db‐id emd‐1832 ‐‐source‐db‐name emdb ‐‐working‐folder temp_working_folder ‐‐db‐path preprocessor/temp/test_db
  VSToolkit requires the presence of Mol* VS database storing entry data (volume, segmentation, metadata, and annotations) that is pre‐constructed using the Preprocessor module. The database is used by VSToolkit as the source of data for CVSX files. It is essentially a directory in a filesystem with subdirectories corresponding to source databases (e.g., emdb, idr) and containing folders that correspond to the individual entries (e.g., emd‐1832). Each entry consists of a ZIP file with volume and segmentation data, and two JSON files: one containing annotations (annotations.json) and another one containing metadata (metadata.json), respectively. In the absence of a pre‐existing database, the Preprocessor module will automatically create one during the initial entry addition process, during which the raw input data (e.g., the volumetric data in a form of a .map file and the segmentation data in a form of an .hff file) are converted in an internally used format, described above.
4 Create query_parameters_emd_1832.json JSON file:
{
"entry_id": "emd‐1832",
   "source_db": "emdb"
}
  Note that this step is different from step 3. In step 3, we performed the preprocessing of the raw input files and added the result to the Mol* VS database, whereas here we create a JSON file with query parameters specifying which data need to be queried from the database by VSToolkit. This protocol demonstrates the utilization of a minimal parameter set, encompassing only the entry ID and the source database name. A comprehensive list of all supported parameters can be found in Table 1.

**Table 1:** VSToolkit Parameters

Parameter	Description	Kind	Type	Default
entry_id	ID of entry in internal database (e.g., emd‐1832)	Mandatory	String	N/A
source_db	Source database (e.g., emdb)	Mandatory	String	N/A
segmentation_kind	Kind of segmentation (e.g., lattice)	Optional	“mesh”, “lattice”, “geometric‐segmentation”	All segmentation kinds
Time	Time frame index	Optional	Integer	All available time frame indices
channel_id	Volume channel ID	Optional	String	All available channel IDs
segmentation_id	Segmentation ID	Optional	String	All available segmentation IDs
max_points	Maximum number of points for volume and/or lattice segmentation. Used to determine the most suitable down sampling level	Optional	Integer	1000000000000

*NOTE*: If an optional parameter is not provided, VSToolkit will use a default value.

**Figure 1:**
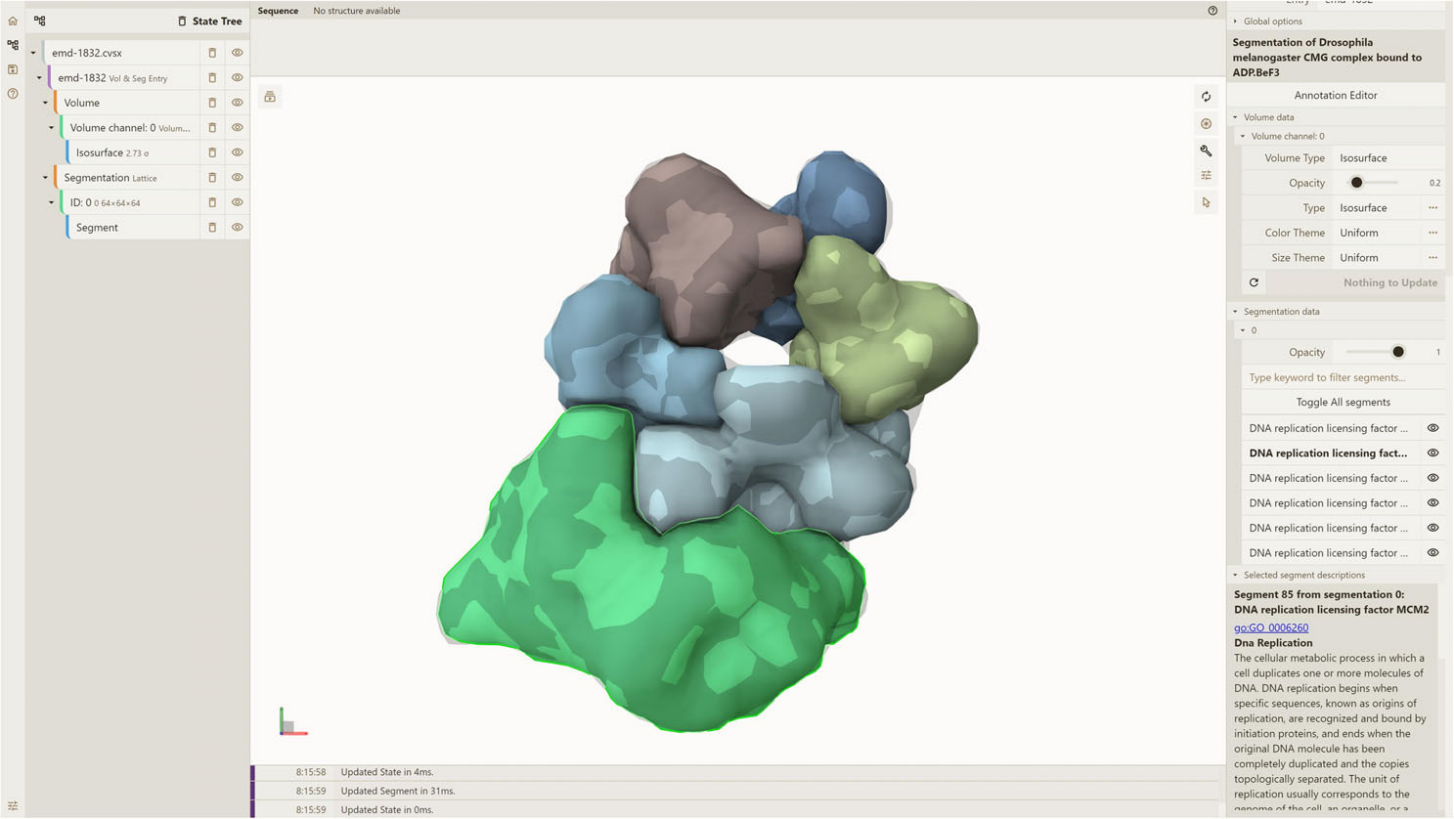
Result of Basic Protocol 1.

5 Use VSToolkit to produce CVSX file:
python vs_toolkit/vs_toolkit.py ‐‐db_path preprocessor/temp/test_db ‐‐out emd‐1832.cvsx ‐‐json‐params‐path query_parameters_emd_1832.json
  To utilize VSToolkit, the user needs to specify the ‐‐db_path argument (path to the Mol* VS database), the ‐‐out argument (output file name, including mandatory .cvsx extension), and the ‐‐json‐params‐path argument (path to the JSON file with query parameters).
  In this protocol, ‐‐db_path is set to preprocessor/temp/test_db to match the ‐‐db_path argument's value used while adding the entry to Mol* VS database on step 2. When executing the command above, VSToolkit retrieves all data available for the specified entry (all available time frames and channels associated with volumetric data, and all available time frames and segmentation types for any included segmentation data) because in query_parameters_emd_1832.json we specified only the entry ID and source database name. These data are packed into the CVSX file (see “Guidelines for understanding results” section for details).
  Note that emd‐1832 entry contains data for a single time frame, a single volume channel, a single segmentation, and one kind of segmentation (corresponding to the source data from EMDB and Volume Browser). However, if an entry contains more channels/segmentations/time frames, all of them would be retrieved.
6 Visualize CVSX file containing a subset of the data queried in the previous step from the Mol* VS database:
  Option A. Drag and drop CVSX file into an instance of Mol* 3D viewer with Mol* VS 2.0 extension (e.g., https://molstar.org/molstar‐volseg/).
  Option B. Use Open Files dropdown menu in the left navigation panel of the user interface of Mol* 3D viewer with Mol* VS 2.0 extension:
  Option C. Use URL https://molstar.org/molstar‐volseg/index.html?cvsx‐url=https://rawcdn.githack.com/molstar/molstar‐volseg/2c677ab0f1b7b7d27f292e0bd209de83e732d216/vs_toolkit/sample_cvsx/emd‐1832.cvsx, linking a Mol* 3D viewer instance with Mol* VS 2.0 extension displaying the data from the pre‐created emd 1832.cvsx file hosted on https://raw.githack.com/.
  Irrespective of the approach used, the resulting visualization within the Mol* 3D viewer shows the volume and segmentation data associated with the EMD‐1832 database entry (see Fig. 1). Notably, the volume data correspond to the EMD‐1832 entry within the EMDB (https://www.ebi.ac.uk/emdb/EMD‐1832), whereas the segmentation data originate from the corresponding SFF file accessible through the Volume Browser (https://www.ebi.ac.uk/empiar/volume‐browser/emd_1832).
  The right navigation panel of the Mol* 3D viewer interface with Mol* VS 2.0 extension displays a filterable list of all segments included within the data. Additionally, upon selection of a specific segment, its associated biologically relevant details (descriptions) are presented within the same panel, below the segment list.

## Basic Protocol 2 VSToolkit—VISUALIZING MULTIPLE TIME FRAMES AND VOLUME CHANNELS

This protocol demonstrates the application of the Volumes & Segmentations Toolkit (VSToolkit) to generate a Cell* Volumes & Segmentations Archive (CVSX) file containing data for all available time frames and channels associated with a designated database entry. This example bears similarities to Basic Protocol 1 in that it showcases fundamental functionalities of VSToolkit, such as creating a CVSX file for a database entry by querying data for all time frames and all channels and visualizing the data. We have chosen this example to emphasize the expanded support for the OME NGFF file format within the current version compared to prior iteration of Mol* Volumes & Segmentations and to demonstrate how a complex dataset with multiple volume channels and time frames can be interactively visualized. Moreover, it provides biological insight into the structure and organization of the cultured human fibroblasts obtained using *in situ* genome sequencing and confocal microscopy (Payne et al., 2021).

### Necessary Resources (also see Basic Protocol 1)

#### Files

Pre‐built database with database entry idr‐13457537 (see Support Protocol 1)

**Figure 2:**
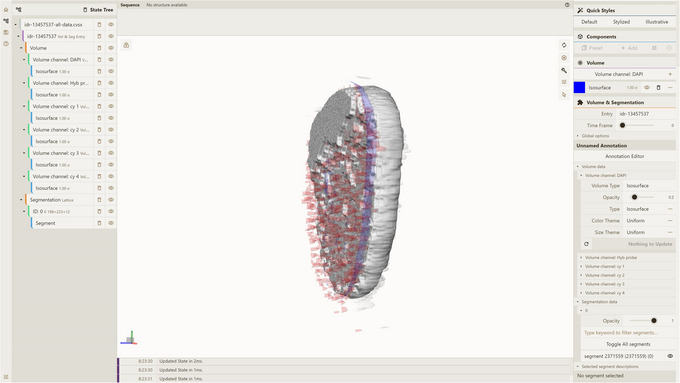
Result of Basic Protocol 2.

1 Install the server; set up and activate the environment.
  Follow step 1 of Basic Protocol 1.
2 Create query‐parameters‐idr‐13457537‐all‐data.json file:
{
   "entry_id": "idr‐13457537",
   "source_db": "idr"
}
3 Use VSToolkit to produce CVSX file:
python vs_toolkit/vs_toolkit.py ‐‐db_path preprocessor/temp/test_db ‐‐out idr‐13457537‐all‐data.cvsx ‐‐json‐params‐path query‐parameters‐idr‐13457537‐all‐data.json
  where preprocessor/temp/test_db is the path to the database used while adding the entry in accordance with Support Protocol 1. This will query all available data for that entry (all time frames and channels for volumes and all time frames and segmentations for all kinds of segmentations). As a result, you should obtain an idr‐13457537‐all‐data.cvsx file.
4 Visualize CVSX file:
  Option A. Drag and drop the idr‐13457537‐all‐data.cvsx file from step 3 into an instance of the Mol* 3D viewer with the Mol* VS 2.0 extension, e.g., https://molstar.org/molstar‐volseg/.
  Option B. Use the Open Files dropdown in the left navigation panel.
  Mol* 3D viewer will recognize the file format and create the visualization, based on CVSX file data, as seen in Figure 2. The resulting view shows volume and segmentation data for the idr‐13457537 Mol* VS database entry, corresponding to image 13457537 from dataset idr0101A (Payne et al., 2021). Note multiple volume channels in the left navigation panel and time‐frame selection slider at the middle right.

## Support Protocol 1 EXAMPLE: ADDITION OF DATABASE ENTRY idr‐13457537

This protocol describes how to add the database entry idr‐13457537 to the Mol* VS database, from which CVSX files are then produced in Basic Protocols 2 and 3. Entry idr‐13457537 contains volumetric and segmentation data in OME NGFF format.

### Necessary Resources

#### Hardware

Internet connection, device capable of supporting Python

#### Software

Python 3.10 (https://www.python.org/downloads/release/python‐3100/), mamba (https://mamba.readthedocs.io/en/latest/installation.html), or conda (https://conda.io/projects/conda/en/latest/user‐guide/install/index.html)

1 Install the server; set up and activate the environment.
  Follow step 1 of Basic Protocol 1 to install the server and perform setup.
2 Add entry to the Mol* VS database.
  a. Create a directory for input data, test‐data/preprocessor/sample_segmentations/idr/idr‐13457537; change the current directory to that; and download the 13457537.zarr folder, which contains the input data in OME NGFF (OME Zarr) format, to the newly created directory using ome_zarr command‐line interface program:
mkdir ‐p test‐data/preprocessor/sample_segmentations/idr/idr‐13457537
cd test‐data/preprocessor/sample_segmentations/idr/idr‐13457537
ome_zarr download https://uk1s3.embassy.ebi.ac.uk/idr/zarr/v0.4/idr0101A/13457537.zarr
  b. Add idr‐13457537 entry:
python preprocessor/cellstar_preprocessor/preprocess.py preprocess ‐‐mode add ‐‐input‐path test‐data/preprocessor/sample_segmentations/idr/idr‐13457537/13457537.zarr ‐‐input‐kind omezarr ‐‐entry‐id idr‐13457537 ‐‐source‐db idr ‐‐source‐db‐id idr‐13457537 ‐‐source‐db‐name idr ‐‐working‐folder temp_working_folder ‐‐db‐path preprocessor/temp/test_db

## Alternate Protocol 1 “Local‐server‐and‐viewer”—VISUALIZING MULTIPLE TIME FRAMES AND VOLUME CHANNELS

This protocol describes how local instances of Mol*3D viewer and server API can be used to visualize data contained in an internal database entry. We have chosen the tubhiswt dataset because it helps to showcase the newly added support for OME‐TIFF‐derived data obtained from custom sources; it further highlights the support for displaying multiple time frames in a single scene. This example is based on an OME‐TIFF series input files and provides biological insights on the structure and organization of tubulin histone GFP coexpressing *C. elegans* embryos (Bembenek et al., 2010).

### Necessary Resources (also see Basic Protocol 1)

#### Files

Pre‐built database with database entry custom‐tubhiswt (see Support Protocol 2)

**Figure 3:**
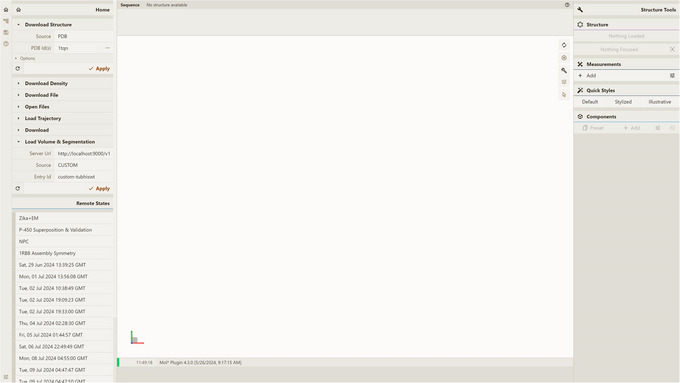
Setup for visualizing database entry custom‐tubhiswt using a local instance of Mol* 3D viewer with the Mol* VS 2.0 extension.

**Figure 4:**
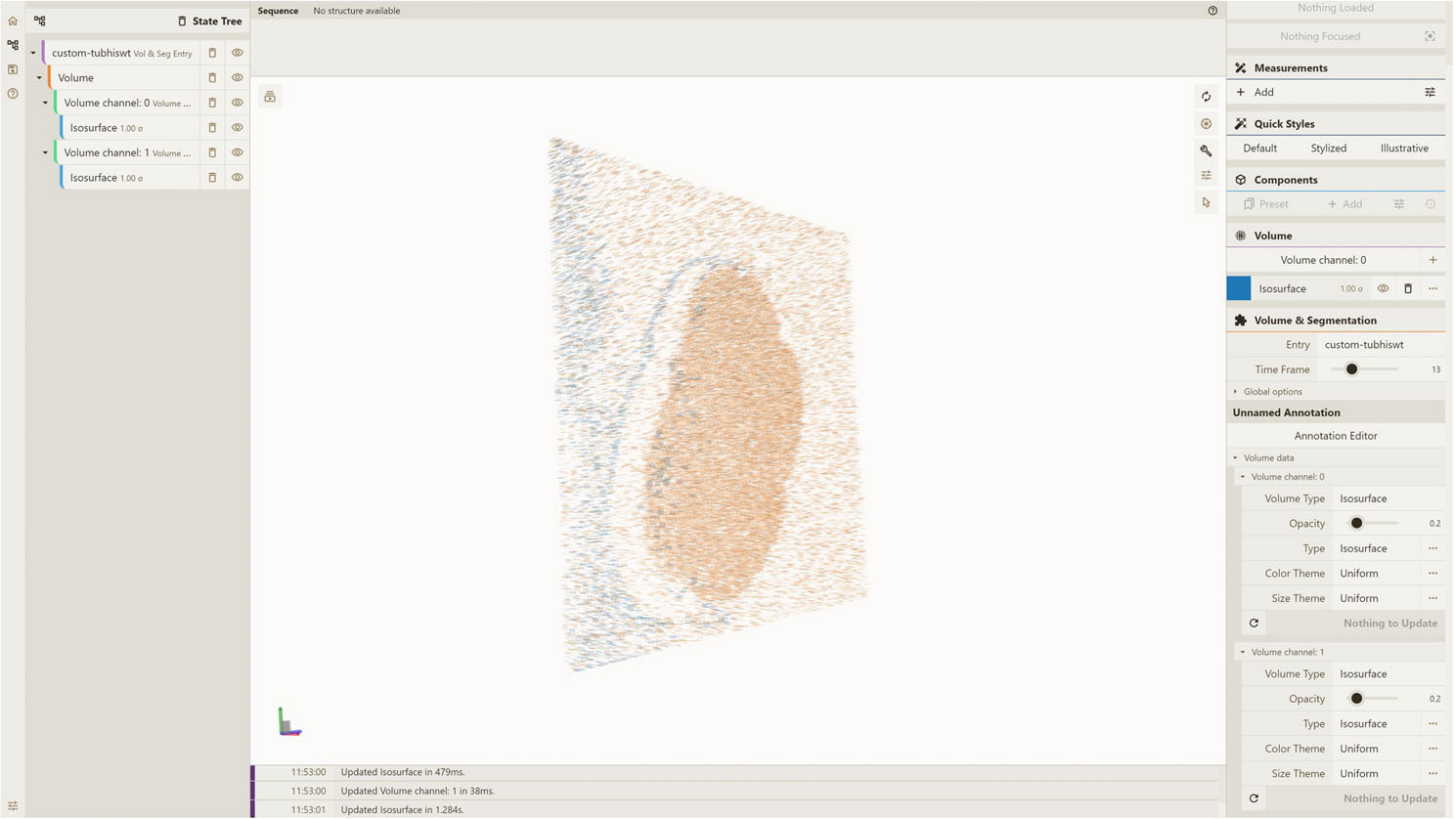
Result of Alternate Protocol 1.

**Figure 5:**
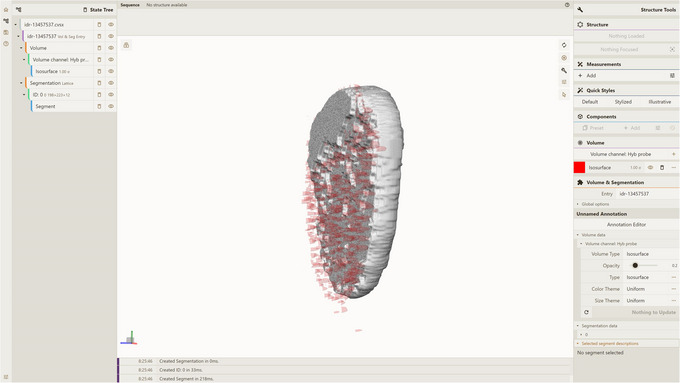
Result of Basic Protocol 3.

1 Install the server; set up and activate the environment.
  Follow step 1 of Basic Protocol 1.
2 Host local instances of server API and Mol* 3D viewer.
  Follow Support Protocols 3 and 4.
3 Visualize a database entry:
  - Open a local instance of Mol* 3D viewer with Mol* VS 2.0 extension in your browser. If you followed Support Protocol 4, it should be available at http://127.0.0.1:8080.
  - Open the Load Volume & Segmentation tab in the left panel, set Server Url in accordance with Support Protocol 3 (by default, http://localhost:9000/v1), and select CUSTOM as Source.
  - Select custom‐tubhiswt in the Entry Id menu (Fig. 3).
  - Click Apply in the left navigation panel to produce the visualization (Fig. 4).
  Note the two volume channels in the left and right navigation panels, as well as a time‐frame slider at right, allowing the user to change the time frame. For Figure 5, we selected the time frame with index 13.

## Support Protocol 2 EXAMPLE: ADDITION OF DATABASE ENTRY CUSTOM‐TUBHISWT

This protocol describes how to add the database entry custom‐tubhiswt to the internal database, which is visualized via local instances of server API and Mol* 3D viewer in Alternate Protocol 1.

### Necessary Resources (also see Support Protocol 1)

#### Software

Zip program or any other program to unzip .zip archives

1 Install the server; set up and activate the environment.
  Follow step 1 from Basic Protocol 1 to install the server and perform the setup.
2 Add entry to the internal database.
  a. Create test‐data/preprocessor/sample_volumes/custom/tubhiswt folder and change current directory to it:
mkdir ‐p test‐data/preprocessor/sample_volumes/custom/custom‐tubhiswt
cd test‐data/preprocessor/sample_volumes/custom/custom‐tubhiswt
  b. Download ZIP archive with the OME‐TIFF series files from OpenMicroscopy website (https://downloads.openmicroscopy.org/images/OME‐TIFF/2016‐06/tubhiswt‐4D.zip) to test‐data/preprocessor/sample_volumes/custom/custom‐tubhiswt
wget https://downloads.openmicroscopy.org/images/OME‐TIFF/2016‐06/tubhiswt‐4D.zip
  c. Unzip the archive into the current directory without creating an archive folder:
unzip ‐j tubhiswt‐4D.zip
  d. Add custom‐tubhiswt entry:
python preprocessor/cellstar_preprocessor/preprocess.py preprocess ‐‐mode add ‐‐input‐path test‐data/preprocessor/sample_volumes/custom/custom‐tubhiswt/tubhiswt_C0_TP0.ome.tif ‐‐input‐kind ometiff_image ‐‐entry‐id custom‐tubhiswt ‐‐source‐db custom ‐‐source‐db‐id custom‐tubhiswt ‐‐source‐db‐name custom ‐‐working‐folder temp_working_folder ‐‐db‐path preprocessor/temp/test_db

## Support Protocol 3 HOSTING Mol* VIEWER WITH THE Mol* VS 2.0 EXTENSION

This protocol describes how to host the local instance of Mol* 3D viewer with Mol* VS 2.0 extension with Volumes & Segmentations extension 2.0, which is used in Alternate Protocols 1 and 2 and Basic Protocol 6 to visualize an internal database entry.

### Necessary Resources

#### Hardware

Internet connection, device capable of supporting a Web browser and WebGL

#### Software

Up‐to‐date Web browser (e.g., Google Chrome, Firefox, Apple Safari) with WebGL support, nodejs, and npm (https://nodejs.org/en/download/package‐manager)

**Figure 6:**
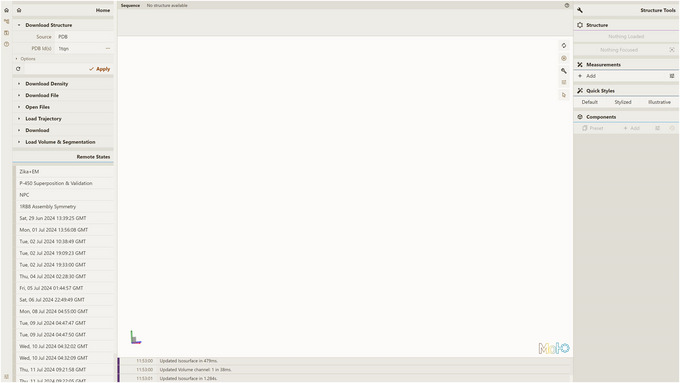
Result of Support Protocol 4: Local instance of Mol* 3D viewer with Mol* VS 2.0 extension.

1 Create a new folder, e.g.:
mkdir molstar‐volseg‐viewer
2 Change directory to that folder:
cd molstar‐volseg‐viewer
3 Initialize new NPM package:
npm init ‐y
4 Install the molstar and molstar‐volseg NPM packages:
npm install molstar
npm install molstar‐volseg
5 Create index.html file in molstar‐volseg‐viewer folder with content identical to that of https://github.com/molstar/molstar‐volseg/blob/master/misc/index.html.
6 Copy molstar.mjs and molstar.css files from molstar‐volseg‐viewer\node_modules\molstar‐volseg\build folder to molstar‐volseg‐viewer folder:
cp node_modules/molstar‐volseg/build/molstar.css.
cp node_modules/molstar‐volseg/build/molstar.mjs.
7 Install http‐server NPM module globally:
npm install http‐server ‐g
8 Serve the index.html webpage using http‐server:
http‐server
  This will serve the webpage with Mol* Viewer with the Mol* Volumes & Segmentations 2.0 extension at http://127.0.0.1:8080 by default (see Fig. 6).

## Support Protocol 4 DEPLOYING SERVER API

This protocol describes how to deploy server API, which is used in Alternate Protocols 1 and 2 and Basic Protocol 6 to visualize an internal database entry.

### Necessary Resources (also see Support Protocol 1)

#### Files

None

1 Install the server; set up and activate the environment.
  Follow step from 1 Basic Protocol 1 to install the server and perform the setup.
2 Host the server API:
python server/cellstar_server/serve.py
  This will deploy server API at http://localhost:9000/v1 with API documentation available at http://localhost:9000/docs.
  Optionally, change DB_PATH constant (line 8 in the code block below) in server/cellstar_server/app/settings.py file to point to the path to your internal database that was built using Preprocessor:
from pathlib import Path
from pydantic import BaseSettings
class _Settings(BaseSettings):
HOST: str = "0.0.0.0"
PORT: int = 9000
DEV_MODE: bool = False
DB_PATH: Path = Path("preprocessor/temp/test_db")
GIT_TAG: str = ""
GIT_SHA: str = ""
settings = _Settings()
  Assuming that you followed one of Support Protocols 1, 4, 5, or 6 while adding entries to the database, it should be set to preprocessor/temp/test_db. Optionally, change the HOST and PORT constants to deploy server API at a local address other than localhost 127.0.0.1 (0.0.0.0) and port other than 9000.

## Basic Protocol 3 VSToolkit—VISUALIZING A SPECIFIC CHANNEL AND TIME FRAME

This protocol describes how the VSToolkit can be used to produce a Cell* Volumes & Segmentations Archive (CVSX) file with data for a specific channel and time frame from the available data for this database entry. This example demonstrates the advanced functionality of the application, such as creating a CVSX file for a database entry by querying data for a specific time frame and channel and visualizing the data. The idr‐13457537 entry was chosen for this as it contains data for multiple time frames and volume channels. Whereas in Basic Protocol 2 and Alternate Protocol 1 all the channels and time frames of this entry were visualized, in this example we focus on a single time frame and channel. In general, this approach can be used to focus the visualization on a specific subset of the data.

### Necessary Resources (also see Basic Protocol 1)

#### Files

Pre‐built database with database entry idr‐13457537 (see Support Protocol 1)

1 Install the server; set up and activate the environment.
  Follow step 1 of Basic Protocol 1.
2 Create JSON file query‐params‐idr‐13457537.json:
{
   "entry_id": "idr‐13457537",
   "source_db": "idr",
   "channel_id": "Hyb probe",
   "time": 4
}
  Note that we are querying only a single volume channel and a single time frame. All the other available data (namely, segmentations) will be queried automatically.
3 Use VSToolkit to produce CVSX file:
python vs_toolkit/vs_toolkit.py ‐‐db_path preprocessor/temp/test_db ‐‐out idr‐13457537.cvsx ‐‐json‐params‐path query‐params‐idr‐13457537.json
  where preprocessor/temp/test_db is the path to the database created according to Support Protocol 1. This will query volumetric data for channel Hyb probe and time frame 4, and segmentation data for all available segmentation kinds and time frame 4, subsequently packing them into the idr‐13457537.cvsx file.
4 Visualize CVSX file:
  Option A. Drag and drop the idr‐13457537.cvsx file from step 3 into an instance of the Mol* 3D viewer with Mol* VS 2.0 extension, e.g., https://molstar.org/molstar‐volseg/.
  Option B. Use the Open Files dropdown in the left navigation panel.
  Option C. Use URL https://molstar.org/molstar‐volseg/index.html?cvsx‐url=https://rawcdn.githack.com/molstar/molstar‐volseg/026803da23fd23e01a967e5f811a09e8e85a7e6d/vs_toolkit/sample_cvsx/idr‐13457537.cvsx to re‐create the view from the file hosted at https://rawcdn.githack.com/molstar/molstar‐volseg/026803da23fd23e01a967e5f811a09e8e85a7e6d/vs_toolkit/sample_cvsx/idr‐13457537.cvsx.
  The resulting view (Fig. 5) shows volume data for channel 5 (Hyb probe) and time frame 4, including segmentation data for time frame 4, both based on the idr‐13457537 Mol* VS database entry. Note that there are only data for a single volume channel (Hyb probe) and a single time frame (4), plus all available segmentation data, in accordance with the query data.

## Basic Protocol 4 VSToolkit—VISUALIZING GEOMETRIC SEGMENTATION

This protocol demonstrates an advanced functionality of the application: creating a CVSX file for a database entry by querying data for a specific segmentation and visualizing the data. We have chosen the EMPIAR 11756 dataset, containing ribosome data, as an example because of its relatively large size, which helps to highlight the support of Mol* VS 2.0 for real‐world large‐scale tomography data as well as the geometric segmentations obtained based on these data. From a technical standpoint, this example is meant to illustrate the ability of the application to visualize a specific segmentation of an entry with multiple segmentations, and showcase the newly added support for geometric segmentations consisting of shape primitives. From a biological perspective, it shows how the user can visualize the dataset containing the imaging data from Chlamydomonas reinhardtii and geometric segmentation showing the position of ribosomes (https://www.ebi.ac.uk/empiar/EMPIAR‐11756/). This approach can be used for the many other tomography‐based EMPIAR entries containing volumetric data along with geometric segmentation data. Furthermore, this feature also have been integrated into the EMPIAR's novel prototype tomogram browser, or “tomoviewer” (https://www.ebi.ac.uk/empiar/tomo‐viewer/; Hatch, 2024), which allows the users to analyze these large entries in a few clicks without having to download them.

### Necessary Resources (also see Basic Protocol 1)

#### Files

Pre‐built database with database entry empiar‐11756 (see Support Protocol 5)

**Figure 7:**
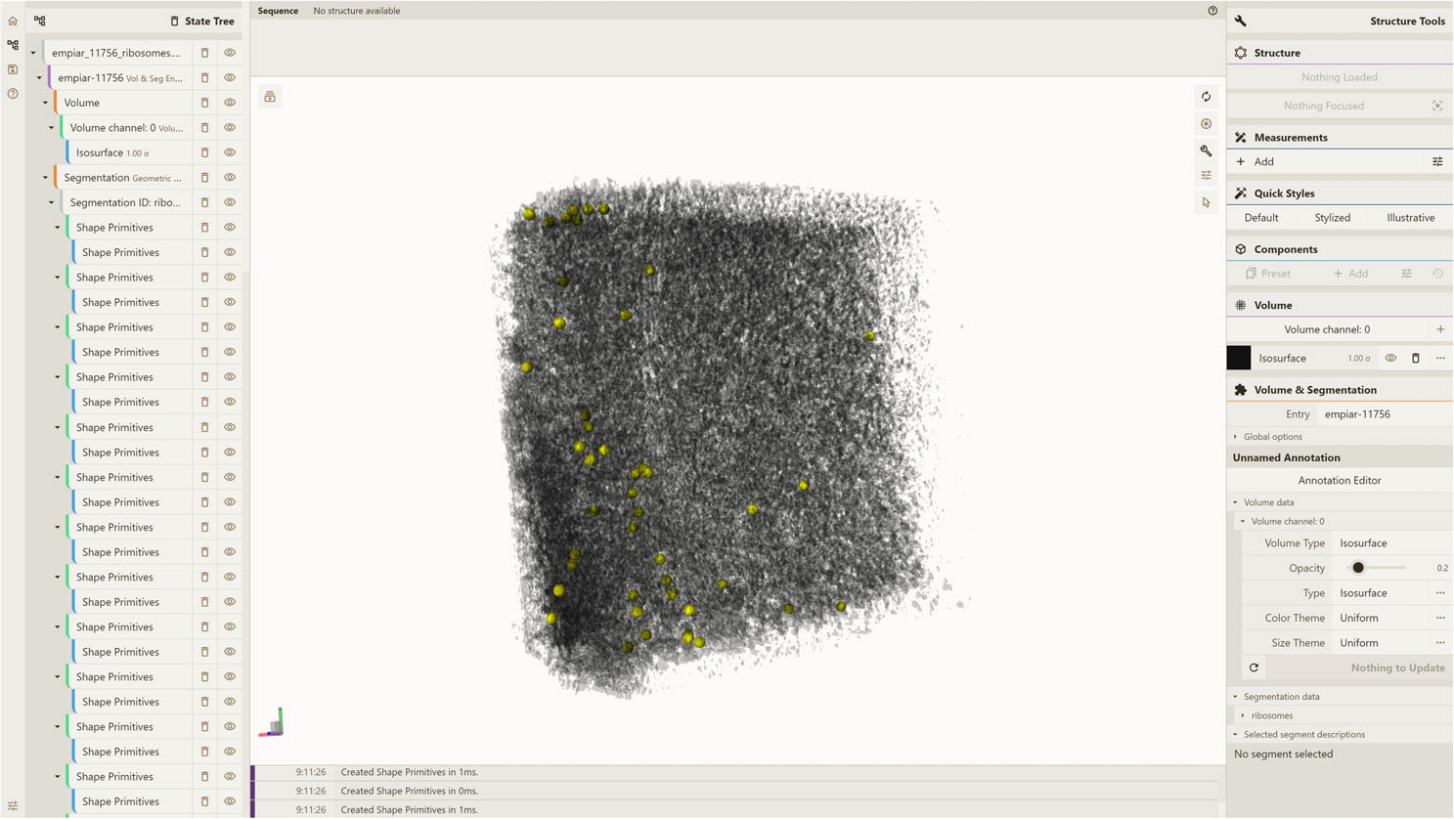
Result of Basic Protocol 4.

1 Install the server; set up and activate the environment.
  Follow step 1 of Basic Protocol 1.
2 Create query_parameters_empiar_11756_ribosomes.json file:
{
  "entry_id": "empiar‐11756",
  "source_db": "empiar",
  "segmentation_kind": "geometric‐segmentation",
  "segmentation_id": "ribosomes",
  "max_points": 10000000
}
  Note that we are querying only one segmentation (segmentation ID ribosomes). We also have to specify max_points (see Table 1) because the size of the original‐resolution volume data is relatively large and we want to have a downsampled version of it in the resulting CVSX file.
3 Use VSToolkit to produce CVSX file:
python vs_toolkit/vs_toolkit.py ‐‐db_path preprocessor/temp/test_db ‐‐out empiar_11756_ribosomes.cvsx ‐‐json‐params‐path query_parameters_empiar_11756_ribosomes.json
  where preprocessor/temp/test_db is the path to the database created according to Support Protocol.
4 This will query all available volume data for that entry and geometric segmentation with segmentation ID ribosomes, packing the results into an empiar_11756_ribosomes.cvsx file.
5 Visualize CVSX file:
  Option A. Drag‐and‐drop the empiar_11756_ribosomes.cvsx file from step 3 into an instance of the Mol* 3D viewer with Mol* VS 2.0 extension, e.g., https://molstar.org/molstar‐volseg/.
  Option B. Use the Open Files dropdown in the left navigation panel.
  The resulting view (Fig. 7) shows volume data together with the segmentation categorized as ribosomes for the empiar‐11756 Mol* VS database entry. This database entry corresponds to a part of EMPIAR‐11756 Electron Microscopy Public Image Archive (EMPIAR) dataset (https://www.ebi.ac.uk/empiar/EMPIAR‐11756/). Specifically, the volume data correspond to one of the available tilt series (17072022_BrnoKrios_Arctis_p3ar_grid_Position_35), and the geometric segmentation was obtained based on preprocessed EMPIAR‐11756 metadata (i.e., particles data from .star files), available on the same web page (see Support Protocol 5).

## Support Protocol 5 EXAMPLE: ADDITION OF DATABASE ENTRY empiar‐11756

This protocol describes how to add the database entry empiar‐11756, which is used in Basic Protocol 4 to produce a CVSX file, to the Mol* VS database. We have chosen the EMPIAR 11756 dataset because of its relatively large size, which helps to highlight the support of Mol* VS 2.0 for real‐world large‐scale tomography data as well as the geometric segmentations obtained based on these data. From a technical standpoint, this example is meant to illustrate the ability of the application to visualize a specific segmentation of an entry with multiple segmentations, while also showcasing the newly added support for visualization of geometric segmentations consisting of shape primitives. From a biological perspective, it shows how the user can visualize the dataset containing the imaging data from *C. reinhardtii* and geometric segmentation showing the position of ribosomes (https://www.ebi.ac.uk/empiar/EMPIAR‐11756/). The approach demonstrated below can be used for the many other tomography‐based EMPIAR entries containing volumetric data along with geometric segmentation data. Furthermore, we have also integrated this approach into the EMPIAR's novel prototype tomogram browser, or “tomoviewer” (https://www.ebi.ac.uk/empiar/tomo‐viewer/; Hatch, 2024), which allows the users to analyze such entries in a few clicks without downloading them.

### Necessary Resources (also see Support Protocol 1)

None

1 Install the server; set up and activate the environment.
  Follow step 1 of Basic Protocol 1 to install the server and perform setup.
2 Add entry to the internal database:
  a. Obtain the raw input files
  i. Create test‐data/preprocessor/sample_volumes/empiar/empiar‐11756 folder, change current directory to it, and download empiar‐11756 electron density map file:
mkdir ‐p test‐data/preprocessor/sample_volumes/empiar/empiar‐11756
cd test‐data/preprocessor/sample_volumes/empiar/empiar‐11756
wget https://ftp.ebi.ac.uk/empiar/world_availability/11756/data/tomoman_minimal_project/cryocare_bin4_tomoname/17072022_BrnoKrios_Arctis_p3ar_grid_Position_35.mrc
  ii. Create test‐data/preprocessor/sample_segmentations/empiar/empiar‐11756 directory, change current directory to it, and download two .star files:
mkdir ‐p test‐data/preprocessor/sample_segmentations/empiar/empiar‐11756
cd test‐data/preprocessor/sample_segmentations/empiar/empiar‐11756
wget https://ftp.ebi.ac.uk/empiar/world_availability/11756/data/tomoman_minimal_project/17072022_BrnoKrios_Arctis_p3ar_grid_Position_35/metadata/particles/rln_nucleosome_bin1_tomo_649.star
wget https://ftp.ebi.ac.uk/empiar/world_availability/11756/data/tomoman_minimal_project/17072022_BrnoKrios_Arctis_p3ar_grid_Position_35/metadata/particles/rln_ribosome_bin1_tomo_649.star
  b. Parse .star files into a format supported by Preprocessor for geometric segmentations:
python preprocessor/cellstar_preprocessor/tools/parse_star_file/parse_single_star_file.py ‐‐star_file_path test‐data/preprocessor/sample_segmentations/empiar/empiar‐11756/rln_ribosome_bin1_tomo_649.star ‐‐geometric_segmentation_input_file_path test‐data/preprocessor/sample_segmentations/empiar/empiar‐11756/geometric_segmentation_input_1.json –sphere_radius 100.0 ‐‐segmentation_id ribosomes ‐‐sphere_color_hex FFFF00 ‐‐pixel_size 7.84 ‐‐star_file_coordinate_divisor 4
python preprocessor/cellstar_preprocessor/tools/parse_star_file/parse_single_star_file.py ‐‐star_file_path test‐data/preprocessor/sample_segmentations/empiar/empiar‐11756/rln_nucleosome_bin1_tomo_649.star ‐‐geometric_segmentation_input_file_path test‐data/preprocessor/sample_segmentations/empiar/empiar‐11756/geometric_segmentation_input_2.json ‐‐sphere_radius 100.0 ‐‐segmentation_id nucleosomes ‐‐sphere_color_hex FF0000 ‐‐pixel_size 7.84 ‐‐star_file_coordinate_divisor 4
    This EMPIAR entry contains relevant data that can be used to render geometric segmentation. These data are stored in .star format, which is not directly supported by Mol* VS 2.0. For the data to be usable, .star files need to be parsed into the standard Mol* VS 2.0 input format for geometric segmentations. This can be achieved by using a custom script preprocessor/cellstar_preprocessor/tools/parse_star_file/parse_single_star_file.py or Jupyter notebook available at https://github.com/molstar/molstar‐volseg/blob/master/preprocessor/cellstar_preprocessor/tools/parse_star_file/parse_single_star_file.ipynb. In parallel, this script makes it possible to set biologically meaningful segmentation IDs for both geometric segmentations based on the data from the EMPIAR entry web page (https://www.ebi.ac.uk/empiar/EMPIAR‐11756/), i.e., ribosomes and nucleosomes).
  c. Create test‐data/preprocessor/sample_volumes/empiar/empiar‐11756/empiar‐11756‐extra‐data.json file to overwrite voxel sizes using extra data Preprocessor functionality:
{
   "volume": {
     "voxel_size": [
       7.84,
       7.84,
       7.84
     ]
   }
}
    The volume map file from EMPIAR entry webpage has the wrong header parameters (the CELLA variable is equal to 0 for all three spatial dimensions). This error translates into the wrong voxel size being automatically determined by Preprocessor based on map header data. To alleviate this problem, above we used the functionality of Preprocessor that allows it to overwrite database entry parameters during preprocessing. Based on the data from EMPIAR entry web page, the voxel size should be 1.96 angstroms for all three dimensions. Because we use volume map file from cryocare_bin4_tomoname folder, this value needs to be multiplied by 4, which yields 7.84 angstroms.
  d. Add empiar‐11756 entry to the internal database:
python preprocessor/cellstar_preprocessor/preprocess.py preprocess ‐‐mode add ‐‐input‐path test‐data/preprocessor/sample_volumes/empiar/empiar‐11756/empiar‐11756‐extra‐data.json ‐‐input‐kind extra_data ‐‐input‐path test‐data/preprocessor/sample_volumes/empiar/empiar‐11756/17072022_BrnoKrios_Arctis_p3ar_grid_Position_35.mrc ‐‐input‐kind map ‐‐input‐path test‐data/preprocessor/sample_segmentations/empiar/empiar‐11756/geometric_segmentation_input_1.json ‐‐input‐kind geometric_segmentation ‐‐input‐path test‐data/preprocessor/sample_segmentations/empiar/empiar‐11756/geometric_segmentation_input_2.json ‐‐input‐kind geometric_segmentation ‐‐entry‐id empiar‐11756 ‐‐source‐db empiar ‐‐source‐db‐id empiar‐11756 ‐‐source‐db‐name empiar ‐‐working‐folder temp_working_folder ‐‐db‐path preprocessor/temp/test_db

## Basic Protocol 5 VSToolkit—VISUALIZING LATTICE SEGMENTATIONS

This protocol describes how the VSToolkit can be used to produce a Cell* Volumes & Segmentations Archive (CVSX) file with visualization data for all available segmentations based on masks of a database entry. We have chosen the EMD‐1273 entry, with data from centrioles and Golgi apparatus, because of the presence of electron‐density masks, which emphasize the Mol* VS 2.0 support for visualizing multiple lattice segmentations in a single scene. Another reason is the relatively large size of the dataset, helping to further highlight the support for large‐scale imagining data. This approach can be applied to other EMDB‐derived entries with lattice segmentations based on electron density masks.

### Necessary Resources (also see Basic Protocol 1)

#### Files

Pre‐built database with database entry emd‐1273 (see Support Protocol 6)

**Figure 8:**
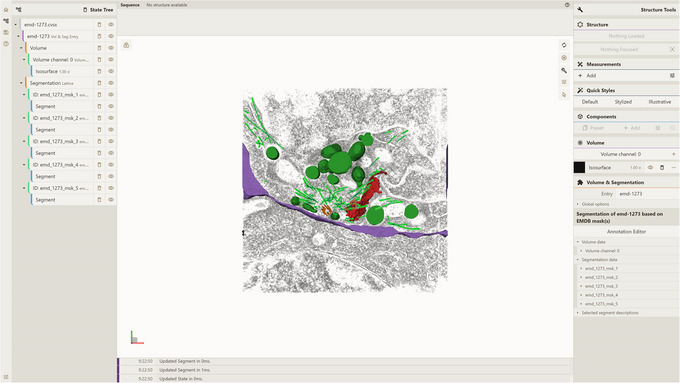
Result of Basic Protocol 5.

1 Install the server; set up and activate the environment.
  Follow step 1 of Basic Protocol 1.
2 Create query_parameters_emd_1273.json JSON file with query parameters:
{
  "entry_id": "emd‐1273",
  "source_db": "emdb",
  "max_points": 100000000
}
  Note that we are querying all available data (volumes and segmentations) for that entry. We also have to specify max_points (see Table 1) because the size of original resolution data is relatively large and we want to have a downsampled version of it in the resulting CVSX file.
3 Use VSToolkit to produce CVSX file:
python vs_toolkit/vs_toolkit.py ‐‐db_path preprocessor/temp/test_db ‐‐out emd‐1273.cvsx ‐‐json‐params‐path query_parameters_emd_1273.json
  where preprocessor/temp/test_db is the path to the Mol* VS database created according to Support Protocol 6. The result will be packed into the file emd‐1273.cvsx.
4 Visualize CVSX file.
  Option A. Drag and drop the emd‐1273.cvsx file from step 3 into an instance of the Mol* 3D viewer with Mol* VS 2.0 extension, e.g., https://molstar.org/molstar‐volseg/.
  Option B. Use the Open Files dropdown in the left navigation panel.
  The resulting view (Fig. 8) shows volume data as well as lattice segmentation data for the emd‐1273 database entry corresponding to volume data for EMDB entry EMD‐1273 (https://www.ebi.ac.uk/emdb/EMD‐1273) and segmentation data based on electron density masks available for that EMDB entry.

## Support Protocol 6 EXAMPLE: ADDITION OF DATABASE ENTRY emd‐1273

This protocol describes how to add the database entry emd‐1273 to the internal database; this is used to produce a CVSX file. The emd‐1273 entry contains volumetric data (electron density maps) and lattice segmentation data (electron density map masks).

### Necessary Resources (also see Support Protocol 1)

#### Files

None

1 Install the server; set up and activate the environment.
  Follow step 1 of Basic Protocol 1 to install the server and perform setup.
2 Add entry to the internal database:
  a. Download electron density map for that entry from EMDB (https://ftp.ebi.ac.uk/pub/databases/emdb/structures/EMD‐1273/map/emd_1273.map.gz) and extract it to test‐data/preprocessor/sample_volumes/emdb/ using gzip (https://www.gnu.org/software/gzip/):
cd test‐data/preprocessor/sample_volumes/emdb/
wget https://ftp.ebi.ac.uk/pub/databases/emdb/structures/EMD‐1273/map/emd_1273.map.gz
gunzip emd_1273.map.gz
  b. Create emdb_masks directory in test‐data/preprocessor/sample_segmentations/ and change current directory to it:
cd test‐data/preprocessor/sample_segmentations/
mkdir emdb_masks
cd emdb_masks
3 Download electron density masks available for EMD‐1273 entry to test‐data/preprocessor/sample_segmentations/emdb_masks/:
wget https://ftp.ebi.ac.uk/pub/databases/emdb/structures/EMD‐1273/masks/emd_1273_msk_1.map
wget https://ftp.ebi.ac.uk/pub/databases/emdb/structures/EMD‐1273/masks/emd_1273_msk_2.map
wget https://ftp.ebi.ac.uk/pub/databases/emdb/structures/EMD‐1273/masks/emd_1273_msk_3.map
wget https://ftp.ebi.ac.uk/pub/databases/emdb/structures/EMD‐1273/masks/emd_1273_msk_4.map
wget https://ftp.ebi.ac.uk/pub/databases/emdb/structures/EMD‐1273/masks/emd_1273_msk_5.map
4 Add emd‐1273 entry:
python preprocessor/cellstar_preprocessor/preprocess.py preprocess ‐‐mode add ‐‐input‐path test‐data/preprocessor/sample_volumes/emdb/emd_1273.map ‐‐input‐kind map ‐‐input‐path test‐data/preprocessor/sample_segmentations/emdb_masks/emd_1273_msk_1.map ‐‐input‐kind mask ‐‐input‐path test‐data/preprocessor/sample_segmentations/emdb_masks/emd_1273_msk_2.map ‐‐input‐kind mask ‐‐input‐path test‐data/preprocessor/sample_segmentations/emdb_masks/emd_1273_msk_3.map ‐‐input‐kind mask ‐‐input‐path test‐data/preprocessor/sample_segmentations/emdb_masks/emd_1273_msk_4.map ‐‐input‐kind mask ‐‐input‐path test‐data/preprocessor/sample_segmentations/emdb_masks/emd_1273_msk_5.map ‐‐input‐kind mask ‐‐entry‐id emd‐1273 ‐‐source‐db emdb ‐‐source‐db‐id emd‐1273 ‐‐source‐db‐name emdb ‐‐working‐folder temp_working_folder ‐‐db‐path preprocessor/temp/test_db
  This will create a database entry with five lattice segmentations based on electron density masks.

## Alternate Protocol 2 “Local‐server‐and‐viewer”—VISUALIZING LATTICE SEGMENTATIONS

This protocol describes how local instances of Mol*3D viewer and server API can be used to visualize data contained in a Mol* VS database entry. It also showcases the support for visualizing multiple lattice segmentations.

### Necessary Resources (also see Basic Protocol 1)

#### Files

Pre‐built database with database entry emd‐1273 (see Support Protocol 6)

**Figure 9:**
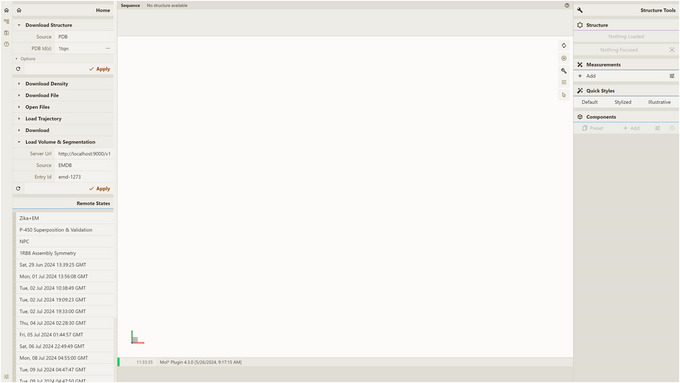
Setup for visualizing database entry emd‐1273 using a local instance of Mol* 3D viewer with the Mol* VS 2.0 extension.

**Figure 10:**
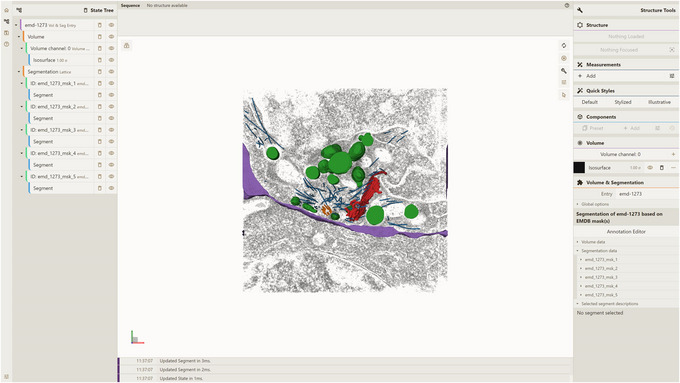
Result of Alternate Protocol 2.

1 Install the server; set up and activate the environment.
  Follow step 1 of Basic Protocol 1.
2 Host local instances of server API and Mol* 3D viewer.
  Follow Support Protocols 3 and 4.
3 Visualize a database entry:
  a. Open a local instance of Mol* 3D viewer with Mol* VS 2.0 extension in your browser. If you followed Support Protocol 4, it should be available at http://127.0.0.1:8080.
  b. Open the Load Volume & Segmentation tab in the left panel, set Server Url in accordance with Support Protocol 3 (http://localhost:9000/v1 by default), and select EMDB as Source.
    After that, the list of Mol* VS database entries for that source database will be available in the Entry Id menu.
  c. Select emd‐1273 in the Entry Id menu (Fig. 9).
  d. Click Apply to produce the visualization (Fig. 10).

4 Edit annotations (optional).
  Part of the annotations, called “description data” for the emd‐1273 database entry, contains placeholder information (e.g., “Segment 1”, “Segment 2”) because segmentation input files (electron density masks) do not contain any biologically relevant information. To add this information manually, please follow Support Protocol 7.

## Support Protocol 7 EDITING ANNOTATIONS

This protocol describes how to edit the value of the “descriptions” key of annotations.json for emd‐1273 database entry; Basic Protocol 5 describes how to produce a CVSX file from that entry.

### Necessary Resources (also see Support Protocol 4)

#### Files

Pre‐built database with database entry emd‐1273 (see Support Protocol 6)

**Figure 11:**
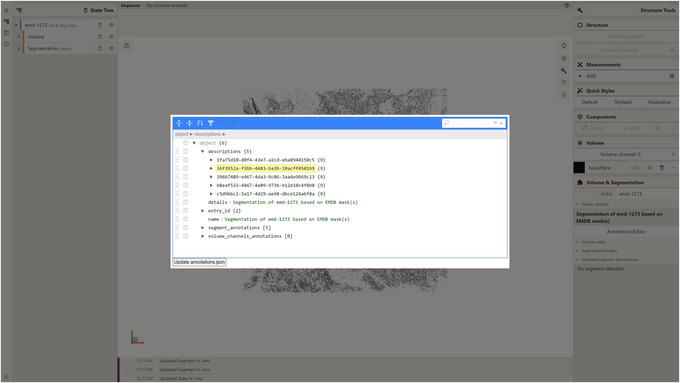
Annotation Editor user interface.

**Figure 12:**
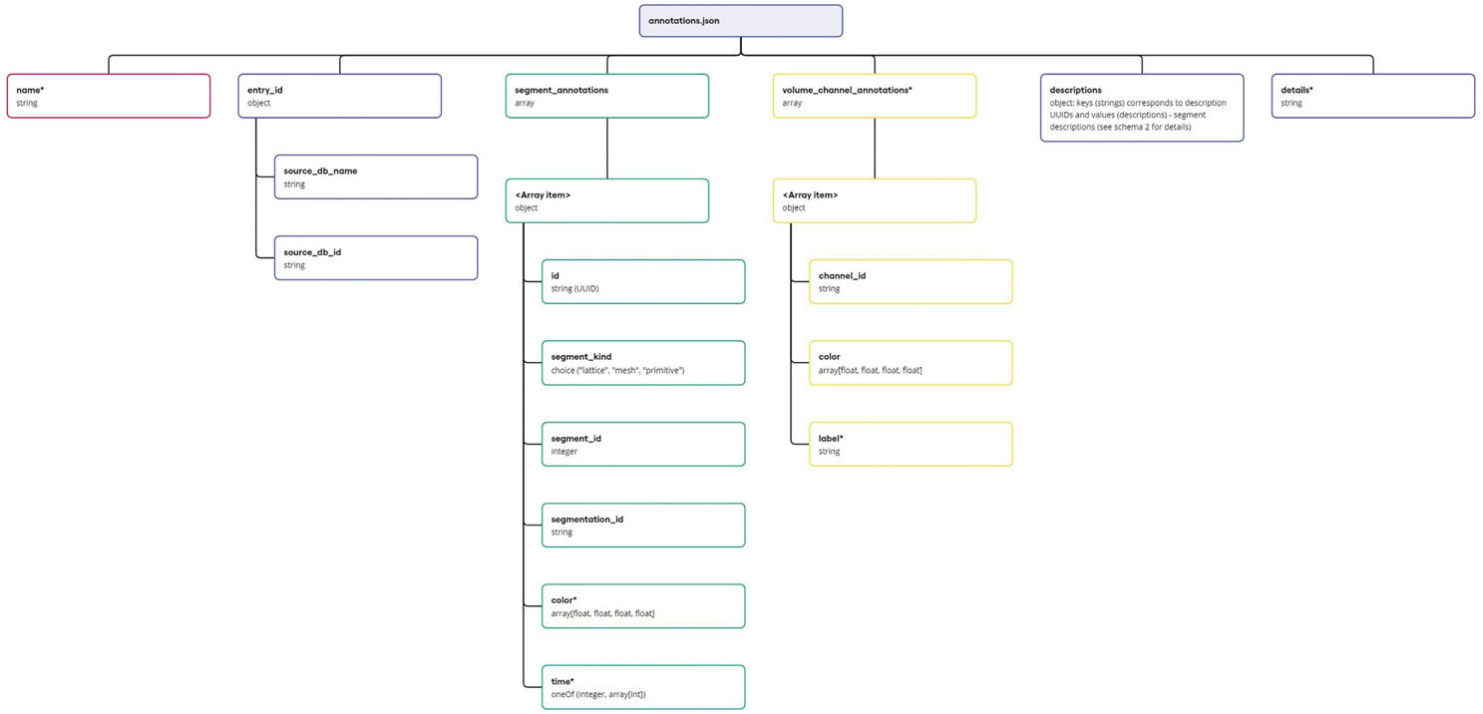
Entry annotations data model.

**Figure 13:**
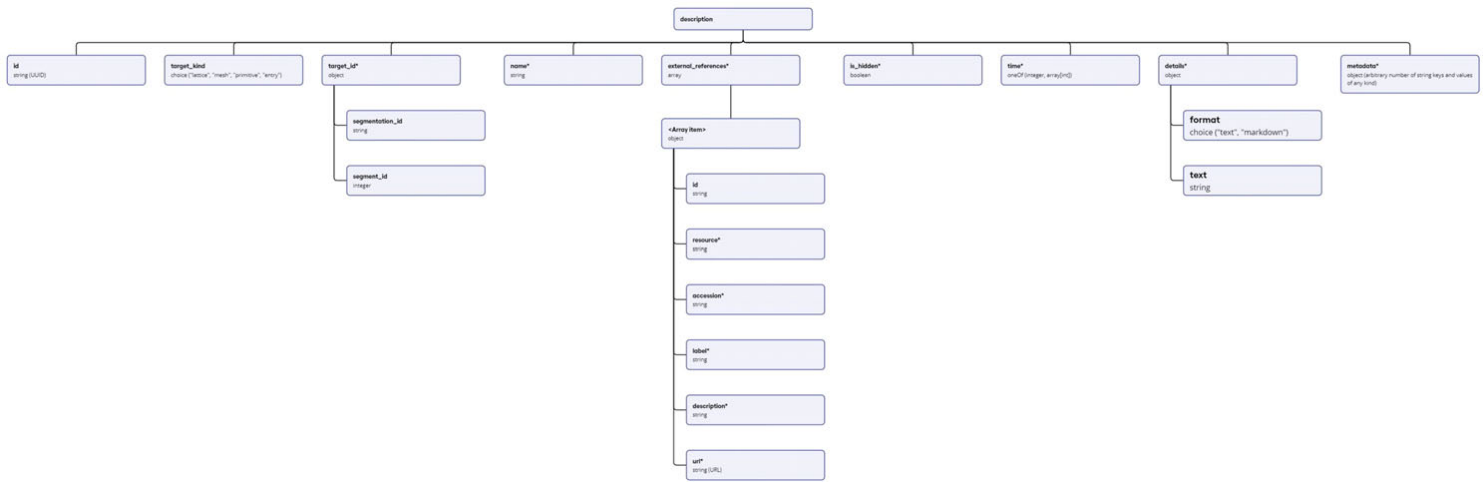
Description data model.

**Figure 14:**
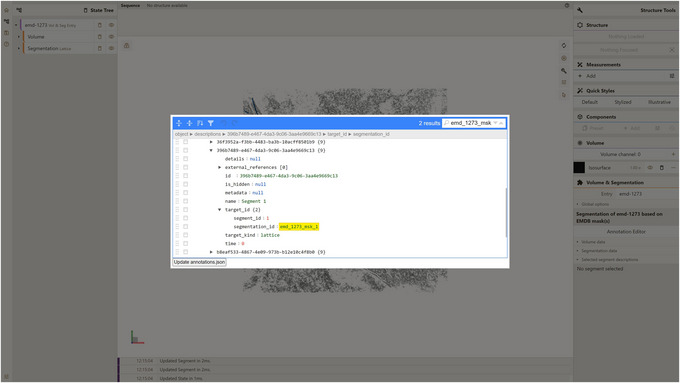
Editing description data for database entry emd‐1273.

**Figure 15:**
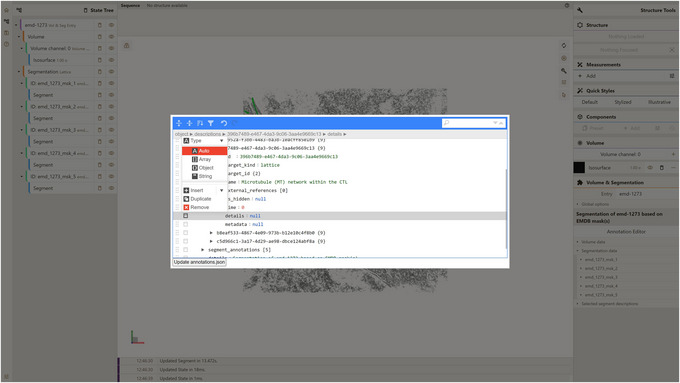
Changing field type in Annotation Editor.

1 Install the server; set up and activate the environment.
  Follow step 1 of Basic Protocol 1 to install the server and perform setup.
2 Host the server API.
  Follow step 2 of Support Protocol 3.
3 Install Mol* and host the local instance of Mol* 3D viewer with Mol* VS 2.0 extension.
  Follow Support Protocol 4 to install Mol* and host the local instance of Mol* 3D viewer with Mol* VS 2.0 extension.
4 Edit description data:
  a. Launch the local instance of the Mol* 3D viewer that was hosted in step 3.
  b. Navigate to the Volumes & Segmentations tab within the left panel of the viewer.
  c. Locate and click the Annotation Editor button positioned at the bottom of the right viewer panel.
    Once this button is clicked, a pop‐up window displaying the Annotation Editor GUI should appear (see Fig. 11). Annotation Editor provides a convenient way to directly edit any part of the file with entry annotation data (annotations.json) hosted as a component of the database entry. Annotation data conform to a specific data model, diagram representations of which are shown in Figures 12 and 13. An example of annotations.json file content can be found online at https://github.com/molstar/molstar‐volseg/blob/master/test‐data/preprocessor/sample_annotations/annotations_example.json, and the complete JSON schema can be found in the Github repository https://github.com/molstar/molstar‐volseg/blob/master/db/cellstar_db/annotations_metadata_schema_2020‐12.json
  d. Enter the search query “emd_1273_msk_1” within the search field in the top right corner of the Annotation Editor.
    In this particular protocol we are interested in editing “descriptions” which represent biologically relevant information related either to an entry as a whole or to individual segments. The emd‐1273 entry within the internal database encompasses five distinct segmentation lattices (named emd_1273_msk_1 through emd_1273_msk_5), each corresponding to one of the five electron density masks used during preprocessing. Each lattice contains a single segment. To leverage the information available on the EMDB entry webpage (https://www.ebi.ac.uk/emdb/EMD‐1273?tab=interpretation) and assign more informative descriptions to these segments, we need to find the descriptions corresponding to each lattice's segment. A basic search functionality integrated into the Annotation Editor GUI (see to Fig. 14) helps users locate keys or values within annotation.json containing all of the search keywords. Note that the keyword “emd_1273_msk_1” can be found in both segment annotation and description, referring to this segment. In this example, we are interested in editing a description for this segment (Fig. 14).
  e. Edit the “name” field of that description, which is filled by placeholder text “Segment 1”.
    Based on the information from https://www.ebi.ac.uk/emdb/EMD‐1273?tab=interpretation, one can fill in this field with the following text, which represents the biologically relevant name for the only segment in the emd_1273_msk_1.map:
Microtubule (MT) network within the CTL
  f. Place mouse pointer on the “details” field of that description, press the button to the left of the field, and change the type of that field to “Object” (Fig. 15).

**Figure 16:**
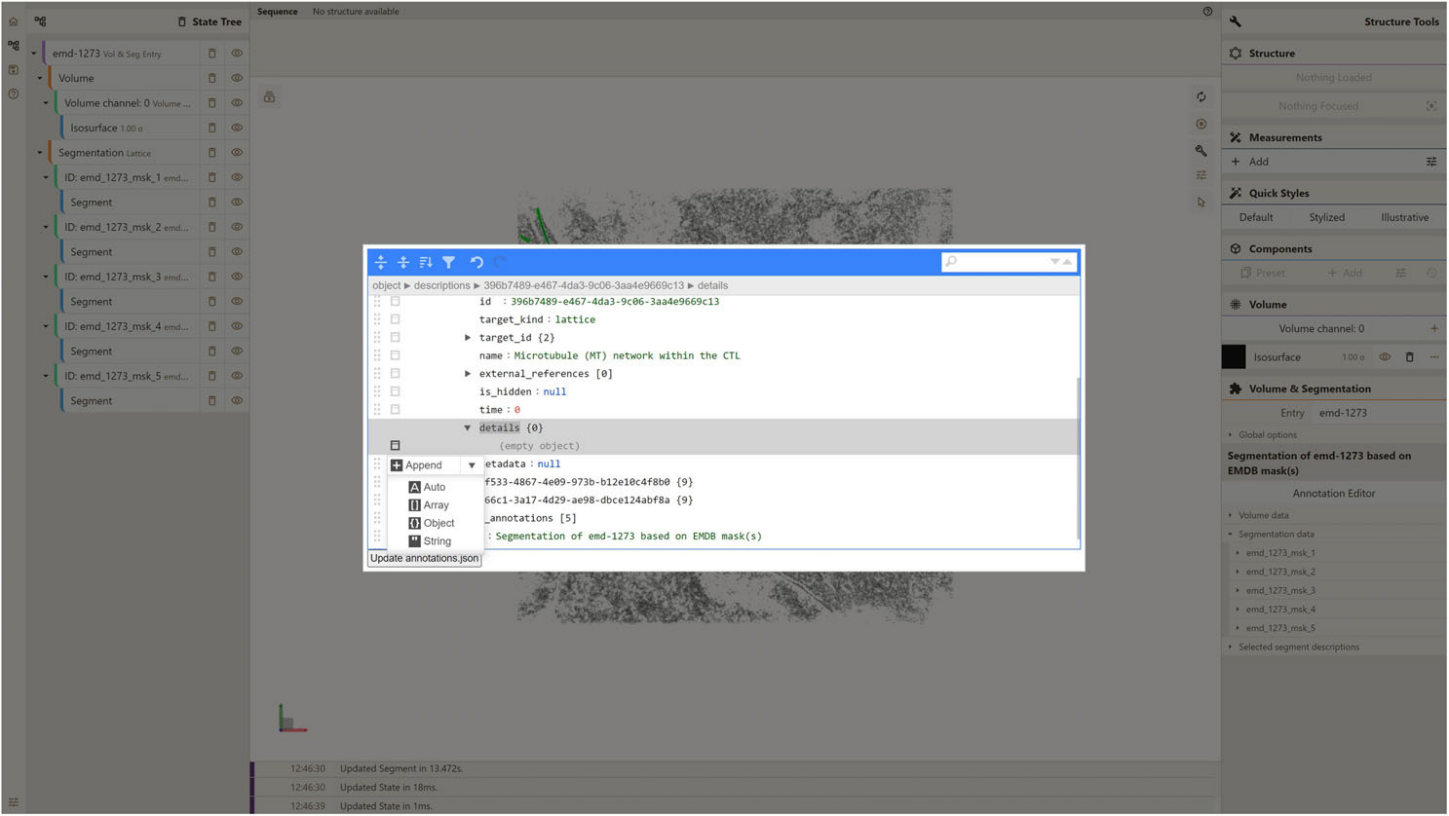
Appending string field in Annotation Editor.

5 Append the string field to the object (Fig. 16).

**Figure 17:**
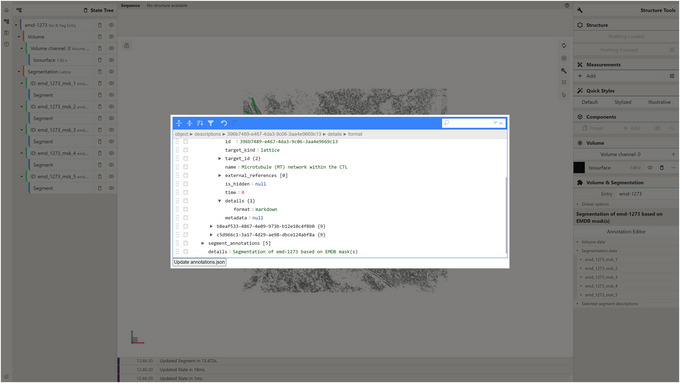
Filling in the name of the field “format”.

6 Fill the key (field) with string “format” and its value with the string “markdown” (Fig. 17).

**Figure 18:**
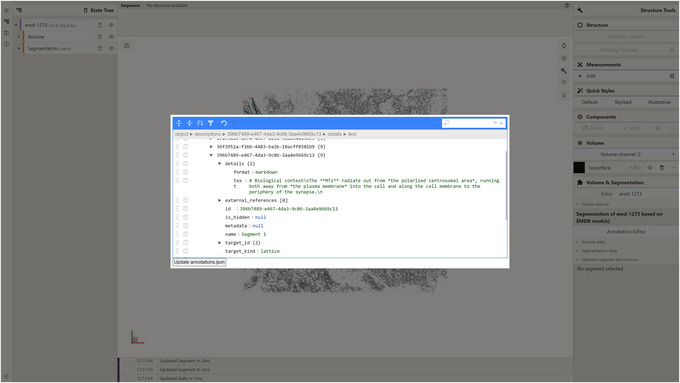
Adding markdown text to the “details” field of a description.

7 Append another string field, and fill in its name (“text”) and value based on the data from the EMDB entry webpage:
# Biological context\nThe **MTs** radiate out from *the polarized centrosomal area*, running both away from *the plasma membrane* into the cell and along the cell membrane to the periphery of the synapse.\n
    As you can see, this supports markdown text, as we specified that in the “format” field (Fig. 18).

**Figure 19:**
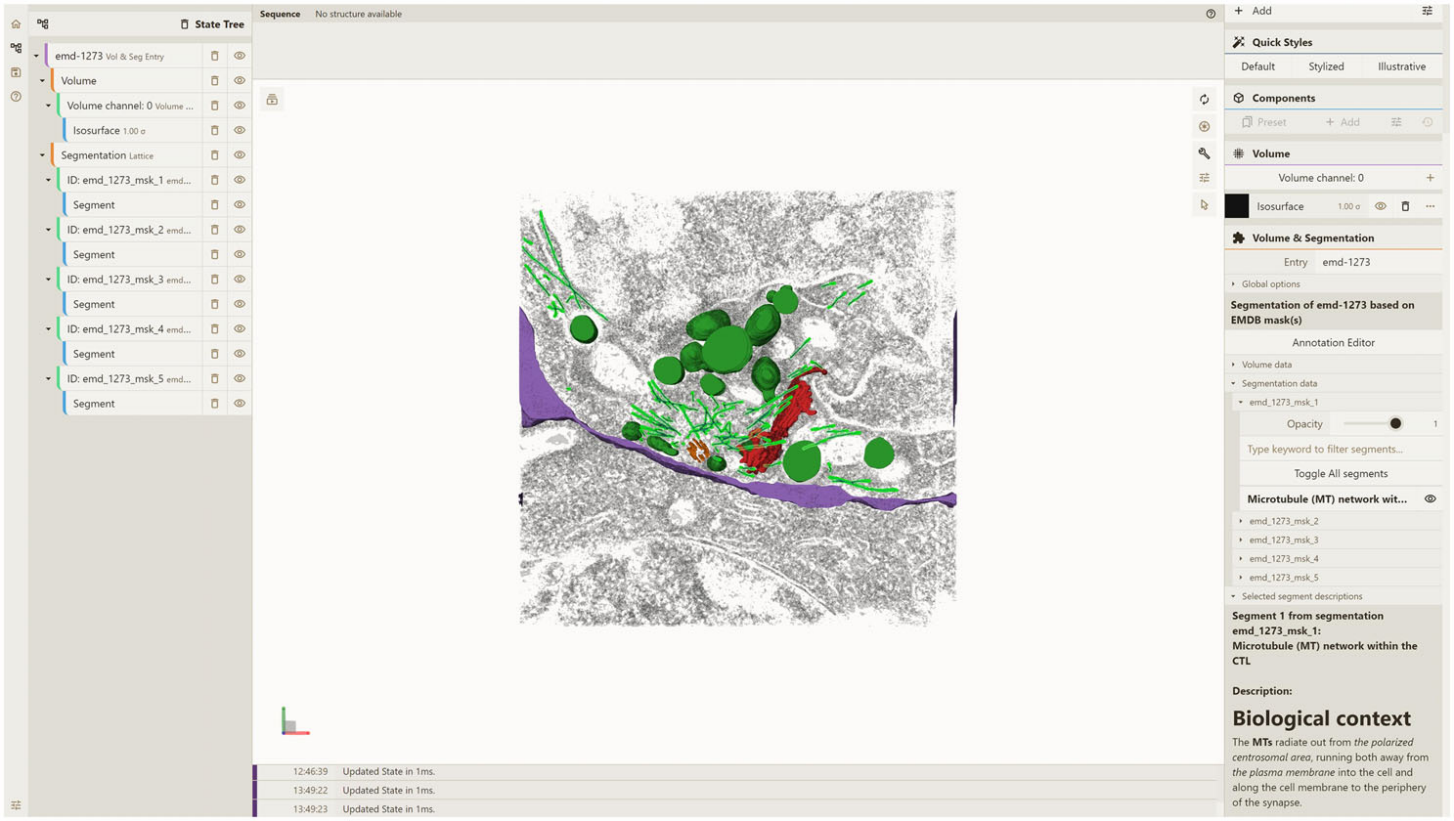
Displaying markdown description text in the Mol* 3D Viewer user interface.

8 Press Update annotations.json button, select the emd_1273_msk_1 segmentation in the right navigation panel, select the Microtubule (MT) network within the CTL segment, and open the Selected segment descriptions dropdown.
  You should see the added markdown text (Fig. 19). Optionally, follow the same approach for the other four masks and fill in the information based on the content of the EMDB entry web page.

## Basic Protocol 6 “Local‐server‐and‐viewer”— VISUALIZING MULTIPLE VOLUME CHANNELS

This protocol describes how local instances of Mol*3D viewer and the server API can be used to visualize data contained in a Mol* VS database entry. We have chosen the idr‐5025553 dataset primarily because it contains large‐scale 2D data (of tonsil sections), which helps to highlight the ability of Mol* VS 2.0 to support data besides 3D data. Additionally, the biological context of this entry (tonsil sections) helps to demonstrate the ability of the application to visualize large‐scale tissue‐level data. This example bears similarities to Alternate Protocols 1 and 2 as it showcases the fundamental functionalities of Mol* VS 2.0, such as visualizing a database entry from a Mol* VS database. In addition, this example further emphasizes the extended support for the OME NGFF file format within the current version compared to prior iteration of Mol* Volumes & Segmentations, along with the support for large‐scale 2D image data.

### Necessary Resources (also see Basic Protocol 1)

#### Files

Pre‐built database with database entry idr‐5025553 (see Support Protocol 8)

**Figure 20:**
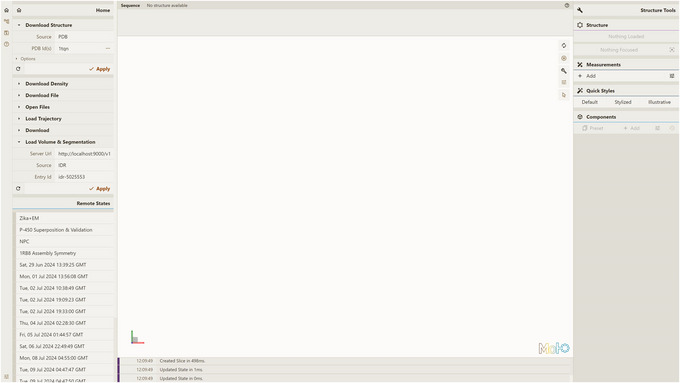
Setup for visualizing database entry idr‐5025553 using a local instance of Mol* 3D viewer with the Mol* VS 2.0 extension.

**Figure 21:**
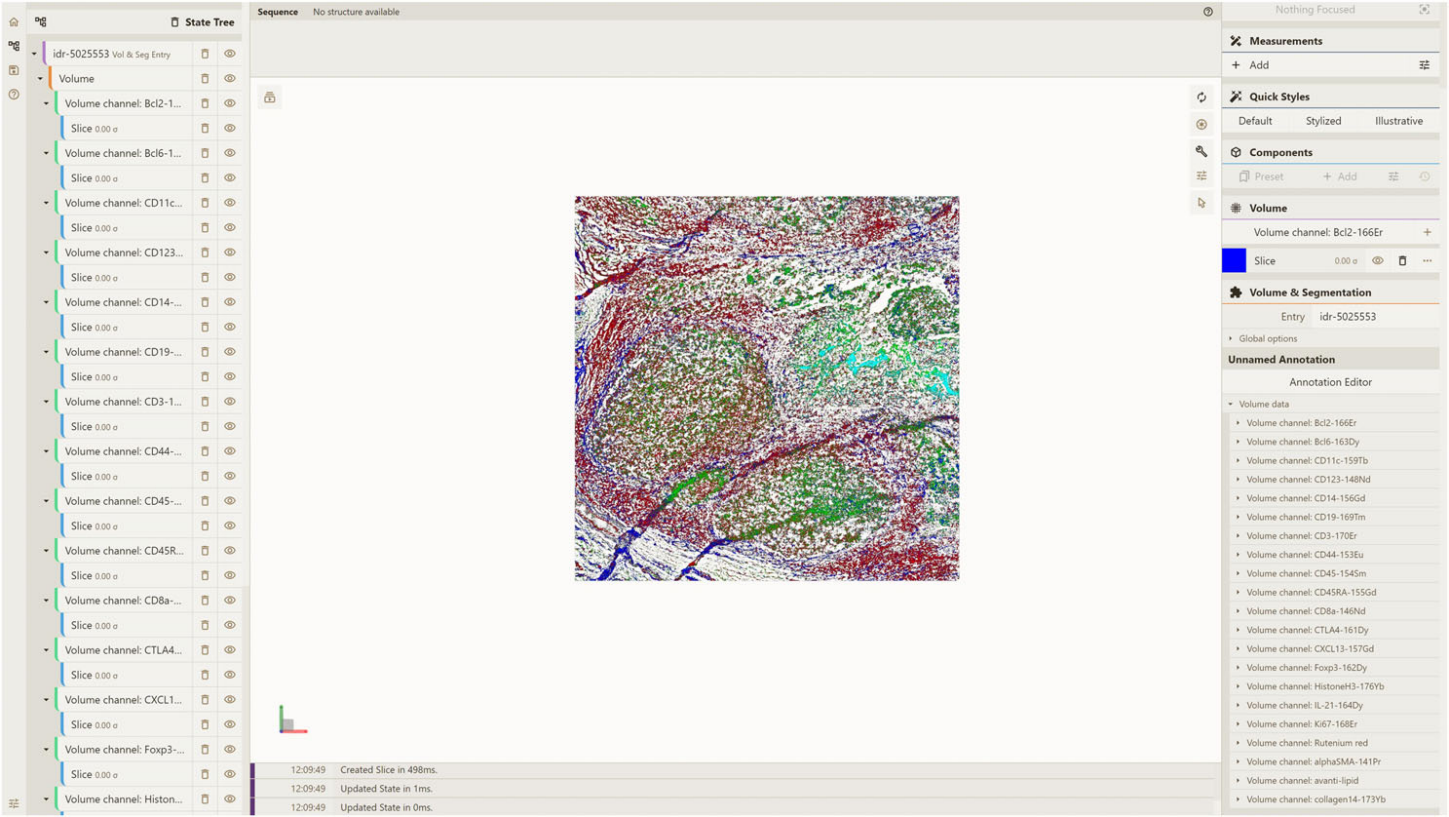
Result of Basic Protocol 6.

1 Install the server; set up and activate the environment.
  Follow step 1 of Basic Protocol 1.
2 Host local instances of server API and Mol* 3D viewer.
  Follow Support Protocols 3 and 4.
3 Visualize a database entry:
  a. Open a local instance of Mol* 3D viewer with Mol* VS 2.0 extension in your browser. If you followed Support Protocol 4, it should be available at http://127.0.0.1:8080.
  b. Open the Load Volume & Segmentation tab in the left panel, set Server Url in accordance with Support Protocol 3 (http://localhost:9000/v1 by default) and select IDR as Source.
  c. Select idr‐5025553 in the Entry Id menu (Fig. 20).
  d. Click Apply to produce the visualization (Fig. 21).
    Note multiple volume channels at the bottom right.

## Support Protocol 8 EXAMPLE: ADDITION OF DATABASE ENTRY idr‐5025553

This protocol describes how to add the database entry idr‐5025553 to the internal database; this is then visualized in Basic Protocol 6 via local instances of server API and Mol* 3D viewer.

### Necessary Resources (also see Support Protocol 1)

#### Files

None

1 Install the server; set up and activate the environment.
  Follow step 1 of Basic Protocol 1 to install the server and perform the setup.
2 Add entry to the internal database.
  a. Create a directory for input data test‐data/preprocessor/sample_segmentations/idr/idr‐5025553 and change current directory to it:
mkdir ‐p test‐data/preprocessor/sample_segmentations/idr/idr‐5025553
cd test‐data/preprocessor/sample_segmentations/idr/idr‐5025553
  b. Download the 5025553.zarr folder with input data in OME NGFF (OME Zarr) format to the newly created directory using ome_zarr command‐line interface program:
ome_zarr download https://uk1s3.embassy.ebi.ac.uk/idr/zarr/v0.4/idr0054A/5025553.zarr
  c. Add idr‐5025553 entry:
python preprocessor/cellstar_preprocessor/preprocess.py preprocess –mode add –input‐path test‐data/preprocessor/sample_segmentations/idr/idr‐5025553/5025553.zarr –input‐kind omezarr –entry‐id idr‐5025553 –source‐db idr –source‐db‐id idr‐5025553 –source‐db‐name idr –working‐folder temp_working_folder –db‐path preprocessor/temp/test_db

## COMMENTARY

### Background Information

This manuscript introduces Mol* Volumes & Segmentations 2.0 (Mol* VS 2.0), the next iteration of a web application for real‐time visualization of large‐scale volumetric and segmentation data together with associated biological annotations. It not only accepts a wider range of input formats than the original Mol*VS (e.g., electron density masks, OME NGFF, OME TIFF) but also seamlessly integrates support for entries with temporal dimensions, multiple volume channels, and various segmentation types (lattice, mesh, geometric). A key addition is VSToolkit, a new tool for generating visualizations from a pre‐built database. Additionally, Mol* VS 2.0 boasts an enhanced user interface, wider input format support, user‐controlled data downsampling procedure, and a revamped data model with separate categories of annotation information for segment rendering and for biological information.

Mol* VS 2.0 offers two user‐friendly methods for visualizing biomacromolecules at various scales. Both require an internal database built by the Preprocessor module. Once the database is built, users can choose the most suitable approach based on their needs.

The first mechanism is implemented within VSToolkit, allowing users to create visualizations based on user‐defined query parameters (see Table 1). This granular control enables the precise specification of the desired part (e.g., specific volume channel, or segmentation) of an internal database entry data that needs to be visualized Upon running, VSToolkit creates Cell* Volumes & Segmentations Archive (CVSX) files, encapsulating the data and additional parameters used to construct 3D visualization of an internal database entry. These CVSX files can then be visualized using any instance of the Mol* 3D viewer with Mol* VS 2.0 extension, such as the publicly available https://molstar.org/molstar‐volseg/. Notably, VSToolkit simplifies the visualization process by eliminating the need to host the local server API and front‐end instances. Furthermore, VSToolkit significantly facilitates the sharing of visualization outputs. CVSX files can be distributed over the Internet or hosted on file‐sharing services. In the latter case, a dedicated URL parameter (cvsx‐url, e.g., https://molstar.org/molstar‐volseg/index.html?cvsx‐url=https://rawcdn.githack.com/molstar/molstar‐volseg/2c677ab0f1b7b7d27f292e0bd209de83e732d216/vs_toolkit/sample_cvsx/emd‐1832.cvsx) can be employed to direct the Mol* 3D viewer to the data source, enabling anyone with the link to access the visualization. Supplementary documentation on the use of VSToolkit can be found online: https://molstar.org/molstar‐volseg/docs/vs_toolkit/overview/

In parallel, Mol* VS 2.0 offers a second visualization mechanism inherited from the initial version of the application. This method involves hosting local instances of the server API and Mol* 3D viewer with the Mol* VS 2.0 extension. Although this approach requires a more complex setup process, it also provides simplified data access through a menu‐driven interface. Users can readily select the source database (e.g., EMDB) and view a corresponding list of available entries. Following the initial setup (internal database construction, server API, and deployment of Mol* 3D viewer with Mol* VS 2.0 extension), users can effortlessly visualize any entry within the internal database with minimal clicks (see Basic Protocol 6 and Alternate Protocols 1 and 2).

The choice between the two visualization mechanisms hinges on user experience and database size. The VSToolkit is particularly well suited to novice users because of its streamlined workflow and simplified sharing capabilities. Conversely, the approach based on hosting the local instances of server API and Mol* 3D viewer presents a more efficient solution for managing and visualizing extensive internal databases. It facilitates effortless switching between different entries and even the simultaneous visualization of multiple entries. The generation of individual CVSX files for a large number of entries using VSToolkit can be cumbersome, making the second approach more practical in such scenarios.

**Figure 22:**
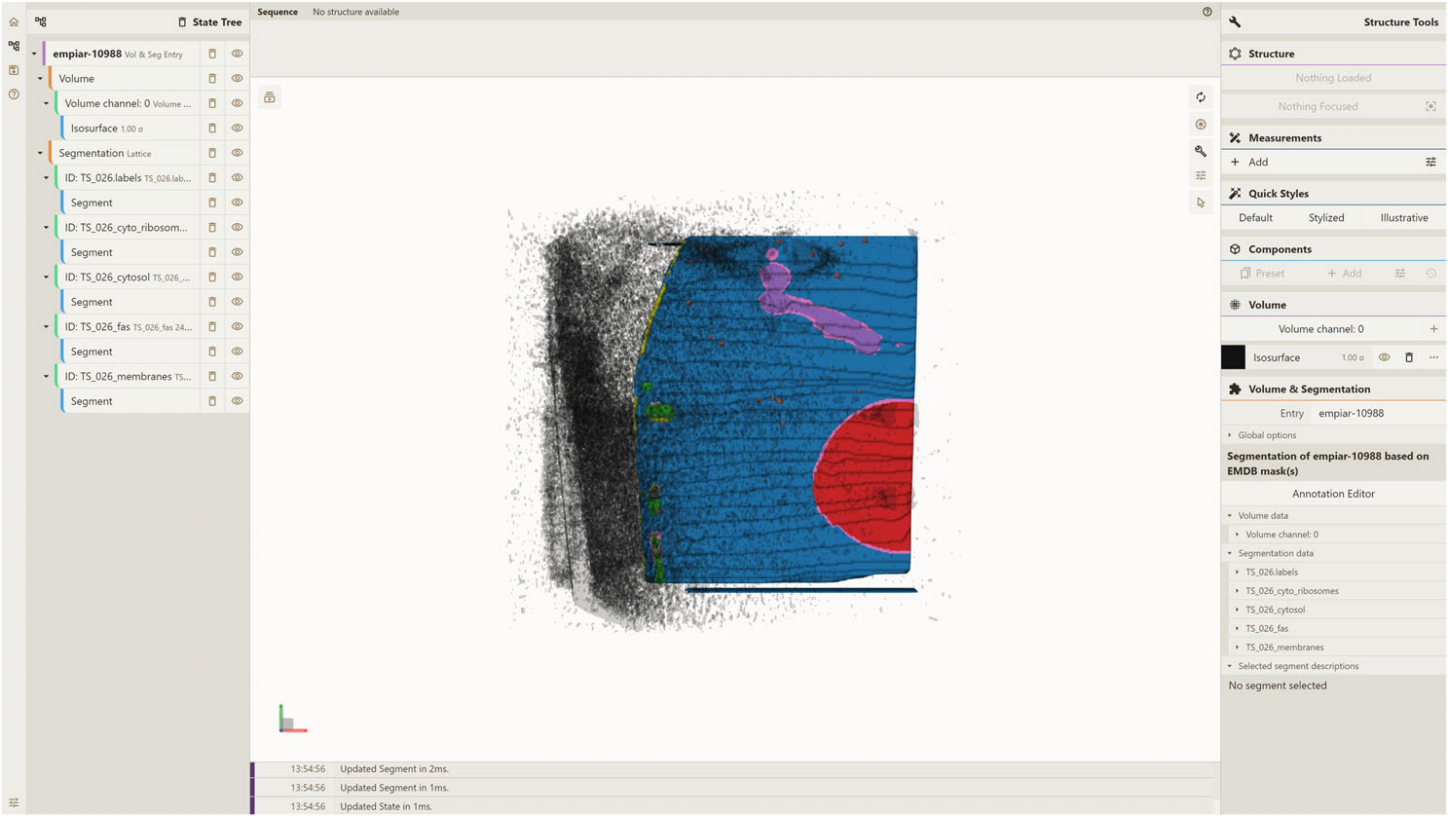
Visualization of database entry empiar‐10988.

Although the protocols in this article provide a comprehensive set of use cases, supplementary examples are also publicly available online. One such example pertains to the EMPIAR‐10988 entry. To preprocess this specific entry, users can refer to the detailed tutorial available online: https://molstar.org/molstar‐volseg/docs/preprocessor/preprocess/#empiar‐10988. To visualize the preprocessed EMPIAR‐10988 entry, users should deploy the server API as (see Support Protocol 3) and host a local instance of the Mol* 3D viewer equipped with the VS 2.0 extension (see Support Protocol 4). Finally, users can proceed by following the steps delineated in Basic Protocol 6 and Alternate Protocols 1 and 2, ensuring the utilization of the appropriate Source (EMPIAR) and Entry Id (empiar‐10988) parameters to produce the visualization (Fig. 22).

**Figure 23:**
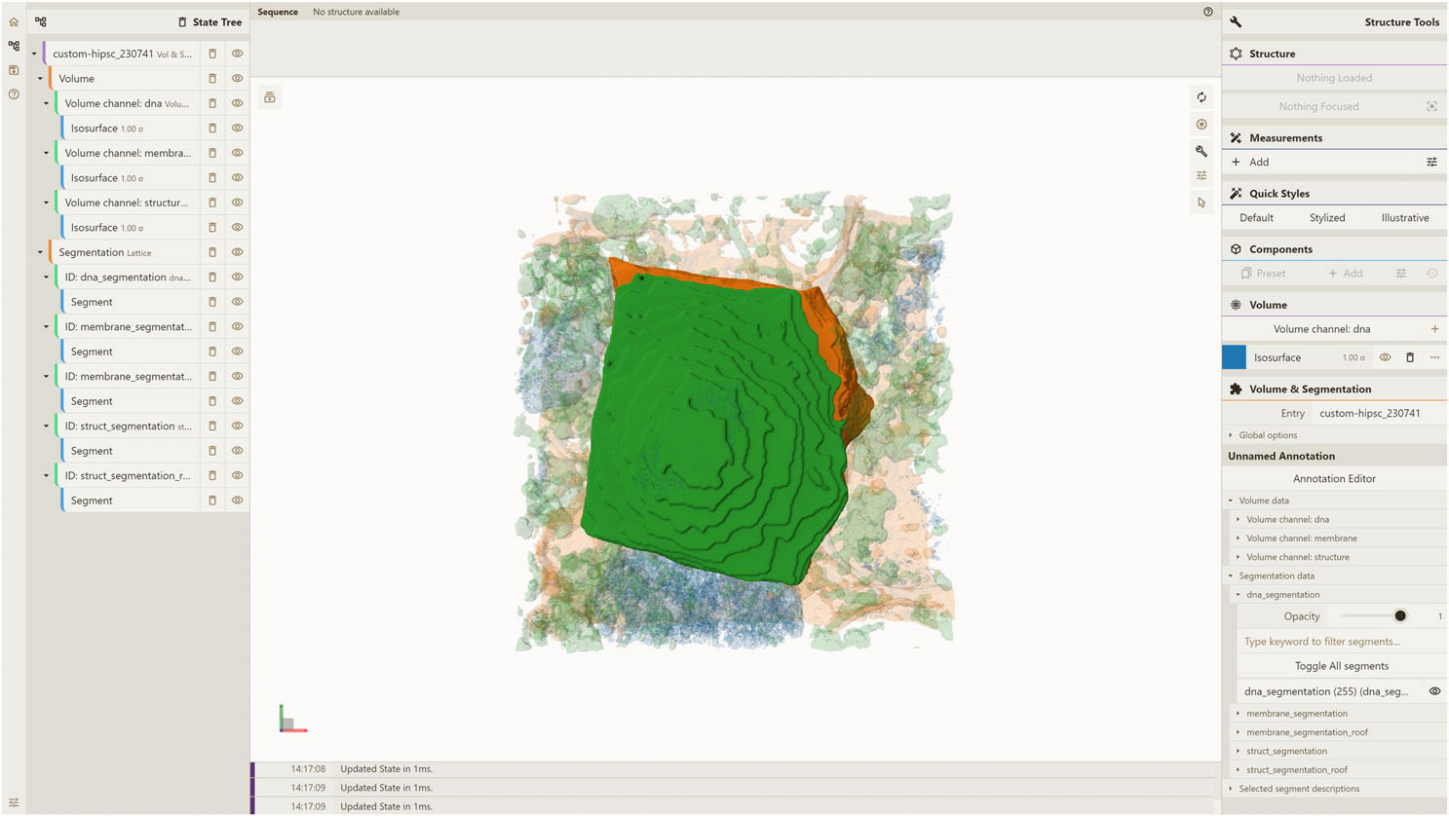
Visualization of database entry custom‐hipsc_230741.

Another illustrative example is based on a part of the human induced pluripotent cell (hiPSC) single‐cell image dataset from the Allen Cell Imaging Collections (https://open.quiltdata.com/b/allencell/tree/aics/hipsc_single_cell_image_dataset/). The specific focus of this use case lies on the preprocessing of a cropped single‐cell raw image (designated “crop_raw”) and its corresponding cropped single‐cell image segmentation (designated “crop_seg”) for a cell with the identifier CellID equal to 230,741. For detailed instructions regarding the preprocessing this specific dataset, please refer to the comprehensive online tutorial available online: https://molstar.org/molstar‐volseg/docs/preprocessor/preprocess/#custom‐hipsc_230741. To visualize this entry (designated custom‐hipsc_230741), users should deploy the server API (see Support Protocol 3), host a local instance of the Mol* 3D viewer with the VS 2.0 extension (see Support Protocol 4), and finally follow the instructions provided in Basic Protocol 6 and Alternate Protocols 1 and 2, being sure to use the appropriate parameters for Source (set to CUSTOM) and Entry Id (set to custom‐hipsc_230741) to generate the desired visualization (Fig. 23).

Beyond the aforementioned visualization mechanisms, Mol* VS 2.0 offers a suite of supplementary tools that expand its functional capabilities. Notably, the Annotation Editor provides a graphical user interface (GUI) for direct editing of annotation data (i.e., the content of annotations.json file) associated with internal database entries. This editor is accessible through the main user interface of the Mol* 3D viewer with Mol* VS 2.0 extension. The Annotation Editor is particularly valuable in cases where the source input files processed by the Preprocessor lack annotation information. In such scenarios, users can leverage the editor to manually incorporate missing annotation data obtained from external resources (refer to Support Protocol 7 for detailed instructions).

Mol* VS 2.0 has been successfully adopted by third‐party resources. An earlier iteration of VSToolkit has been integrated into EMPIAR's novel prototype tomogram browser for cryo‐electron tomography (cryo‐ET) data visualization (https://www.ebi.ac.uk/empiar/tomo‐viewer/; Hatch, 2024). This integration leverages VSToolkit's functionality to generate static visualization files based on tomogram data and corresponding geometric segmentation input. Subsequently, the Mol* 3D Viewer is employed to render the files produced by VSToolkit. Notably, this visualization approach facilitates the identification of particle locations within the tomogram data. These particles, corresponding to various organelles such as ribosomes, microtubules, or nucleosomes, are represented as spheres within the geometric segmentation data. The incorporation of VSToolkit not only streamlines the exploration of intricate cellular structures but also enhances the accessibility of high‐resolution cryo‐ET imaging data.

### Critical Parameters

#### Visualization with the VSToolkit

‐‐db_path parameter defines the path to the internal database from which the data will be queried, ‐‐out parameter specifies the output file path (with the mandatory .cvsx extension) where the retrieved data will be stored, and ‐‐json‐params‐path parameter indicates the path to the JSON file containing the user‐defined query parameters. A minimal set of required query parameters for VSToolkit that should be specified in the JSON file includes entry_id and source_db. When only these parameters are provided, VSToolkit retrieves all data available for the specified entry within the internal database. These include volume data for all available channels and time frames, as well as segmentation data encompassing all available time frames and all types of segmentations. A comprehensive list of all supported parameters can be found in Table 1.

#### Visualization with the “local‐server‐and‐viewer” approach

DB_PATH parameter from server/cellstar_server/app/settings.py file defines the path to the internal database, constructed with Preprocessor module. It is crucial to ensure that DB_PATH accurately points to the location of the user's internal database. The Server Url parameter is associated with the locally hosted instance of the Mol* 3D Viewer. A menu for selecting the value of this parameter is located in the left panel under the Load Volume & Segmentation tab. The parameter's value should correspond to the URL where the local instance API can be accessed. By default, this URL is http://localhost:9000/v1.

#### Advanced parameters

The Preprocessor offers a selection of additional command‐line arguments that provide more control over the preprocessing workflow. These arguments are not mandatory but can be used to tailor the preprocessing to specific requirements. Detailed description of each argument is available online at https://molstar.org/molstar‐volseg/docs/preprocessor/advanced/.

In addition to the standard command‐line arguments discussed previously, the Preprocessor offers a set of advanced functionalities currently classified as experimental. These functionalities are designed to supplement database entries (i.e., incorporate supplementary data into the corresponding database entry during the preprocessing stage) or overwrite database entry parameters, which is valuable for correcting inconsistencies or inaccuracies within the raw input data. Note that this functionality is under active development, and we warmly welcome any suggestions or contributions from the user community to further enhance it.

**Figure 24:**
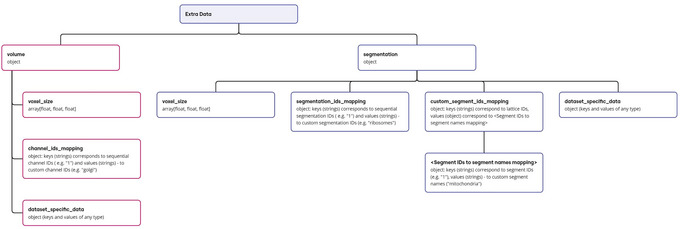
Extra data model.

To leverage this functionality, users should provide an additional input file in JSON format alongside the standard input files containing volume and/or segmentation data. The structure of this JSON file needs to adhere to a specific data model (Fig. 24). It is noteworthy that all fields within the extra data section are designated as optional. In addition, the file containing the extra data must be provided as the first input file during the preprocessing execution. The full JSON schema is available online at GitHub repository: https://github.com/molstar/molstar‐volseg/blob/master/db/cellstar_db/extra_data_schema.json.

Support Protocol 5 provides a practical example of utilizing JSON with extra data as one of the Preprocessor's input arguments. In this specific instance, this advanced functionality is employed to override the voxel size that the Preprocessor would automatically determine based on erroneous map header data within the volume map input file.

The following excerpt showcases a sample structure for a JSON file containing extra data, designed for the aforementioned use case of overriding voxel size information:

{
   "volume": {
     "voxel_size": [
      7.84,
      7.84,
      7.84
    ]
  }
}

Another illustrative example pertains to the preprocessing workflow for the EMPIAR‐10988 entry. A detailed tutorial outlining this process can be found online at https://molstar.org/molstar‐volseg/docs/preprocessor/preprocess/#empiar‐10988. In this case, the purpose of using extra data is to modify the segment IDs automatically assigned by the Preprocessor during the preprocessing of electron density mask files. Grid data in these files typically contain integer values, e.g., “1”, “2”, etc., corresponding to segment IDs. The Preprocessor automatically uses these values to assign generic segment names, such as “Segment 1”, “Segment 2”, etc. Although it is possible to manually edit this via Annotation Editor (see Support Protocol 7), we offer an alternative approach involving extra data functionality that allows the user to achieve the same result with less manual effort. Following this method, these generic segment IDs can be replaced with more biologically relevant segment names, such as “cytoplasm” or “mitochondria”, by providing a JSON file following a specific format as the first input file to the Preprocessor.

The following code snippet illustrates a possible configuration for the extra‐data JSON file, specifically designed to achieve the aforementioned customization of segment names for the EMPIAR‐10988 entry:

{
   "segmentation": {
     "custom_segment_ids_mapping": {
       "TS_026.labels": {
        "1": "cytoplasm",
        "2": "mitochondria",
        "3": "vesicle",
        "4": "tube",
        "5": "ER",
        "6": "nuclear envelope",
        "7": "nucleus",
        "8": "vacuole",
        "9": "lipid droplet",
        "10": "golgi",
        "11": "vesicular body",
        "13": "not identified compartment"
       }
    }
   }
}

Although Preprocessor is typically used to add entries to the internal database (“preprocess” command), it can also be used for other purposes, such as removing entries from the database. For comprehensive reference, a detailed list of all supported Preprocessor commands with descriptions is provided at https://molstar.org/molstar‐volseg/docs/, “Preprocessor documentation” tab in the left panel, see sections of individual commands for details.

### Troubleshooting

Table 2 provides an overview of potential issues that users may encounter when employing the outlined protocols, together with possible underlying causes and corresponding solutions. For any inquiries seeking further clarification or to report errors encountered during protocol execution, users are encouraged to submit an issue on the Mol* Volumes & Segmentations code repository hosted on GitHub (https://github.com/molstar/molstar‐volseg/issues).

**Table 2:** Potential Issues, Possible Causes, and Solutions

Problem	Possible cause	Solution
Python script cannot be executed	Dependency on Python 3.10	Install server using Python interpreter 3.10 or higher.
File parsing error while trying to open CVSX file in Mol* 3D viewer	Trying to use unsupported file format	Use supported file format (CVSX). Check that the extension (.cvsx) matches actual file format.
Error while trying to open CVSX file hosted at file hosting service in Mol* 3D viewer	Wrong file URL	Check if the viewer tries to fetch the CVSX file from the correct location.
	File‐hosting service does not support CORS	Ensure the file hosting service supports CORS (Cross‐Origin Resource Sharing) header, AS otherwise the browser will block the request.

### Understanding Results

Mol* Volumes & Segmentations 2.0 (Mol* VS 2.0) facilitates the visualization of volumetric, segmentation, and annotation data derived from various sources. For a comprehensive list of the supported features, please refer to the documentation at https://molstar.org/molstar‐volseg/docs/. Below we outline the overall workflow and highlight key aspects.

The initial and critical stage within the visualization pipeline involves the establishment of an internal (Mol* VS 2.0) database. This internal database functions as a repository for entry data generated by the Preprocessor module, a server‐side component. An internal database is a directory in a filesystem. Subdirectories of this directory correspond to source databases (e.g., EMDB, IDR). Each subdirectory contains folders that correspond to the individual entries (e.g., emd 1832). Each entry comprises a ZIP file with volume and segmentation data, along with two JSON files: one with the annotations (annotations.json) and the other with the metadata (metadata.json). Following the construction of the internal database, data visualization can be achieved through two primary approaches.

• **“Local‐server‐and‐viewer**,” which entails hosting local instances of both the server API and the Mol* 3D viewer with Mol* VS 2.0 extension. Data visualization within the Mol* 3D viewer is then facilitated by selecting the appropriate source database (Source) and Entry Id in the left panel under the Load Volume & Segmentation tab, as detailed in Basic Protocol 6 and Alternate Protocols 2 and 5.
• **VSToolkit**, which allows users to generate CVSX files by querying the internal database to retrieve the desired data. These files then can be visualized within any instance of the Mol* 3D viewer with Mol* VS 2.0 extension (see Basic Protocols 1‐5).

CVSX files, generated by the VSToolkit, contains a user‐requested subset of data pertaining to a specific entry within the internal database. This data subset can encompass volumetric data, segmentation data, and annotation data, along with associated metadata. Furthermore, CVSX files incorporate two additional JSON files: index.json detailing the contents of the CVSX file, and query.json, documenting query parameters used to generate the CVSX file.

A CVSX file, from a technical standpoint, is organized identically to a standard ZIP archive. The .cvsx extension serves as a distinct marker to differentiate these files from their non‐specialized counterparts, although their underlying structural organization remains identical. Notably, the Mol* 3D Viewer (Sehnal et al., 2021), a well‐established application for 3D molecular graphics visualization, offers support for CVSX files via a dedicated extension available at NPM (https://www.npmjs.com/package/molstar‐volseg), with source code available at https://github.com/molstar/molstar‐volseg/blob/master/molstar‐extension. The extension can parse the contents of CVSX files, enabling the rendering of volumetric and segmentation data alongside any associated annotations. Online instance of Mol* 3D Viewer is available at https://molstar.org/molstar‐volseg/.

### Author Contributions

**Aliaksei Chareshneu**: Data curation; investigation; methodology; software; visualization; writing—original draft; writing—review and editing. **Alessio Cantara**: Data curation; validation; writing—original draft; writing—review and editing. **Dominick Tichý**: Software; writing—original draft; writing—review and editing. **David Sehnal**: Conceptualization; funding acquisition; methodology; project administration; resources; software; supervision; validation; writing—review and editing.

### Conflict of Interest

The authors declare no conflict of interest.

## Data Availability

The source code for Mol* VS 2.0 is publicly available on GitHub (https://github.com/molstar/molstar‐volseg), fostering transparent development and encouraging community contributions. This repository serves as a platform for users to report issues, propose enhancements, and actively participate in the project's evolution. Mol* VS 2.0 functionality is seamlessly integrated into Mol* as an extension (https://github.com/molstar/molstar‐volseg/tree/master/molstar‐extension), online instance of which is available at https://molstar.org/molstar‐volseg/. This extension is also distributed through the NPM package manager (https://www.npmjs.com/package/molstar‐volseg).
